# New insights into the transposition mechanisms of IS*6110* and its dynamic distribution between *Mycobacterium tuberculosis* Complex lineages

**DOI:** 10.1371/journal.pgen.1007282

**Published:** 2018-04-12

**Authors:** Jesús Gonzalo-Asensio, Irene Pérez, Nacho Aguiló, Santiago Uranga, Ana Picó, Carlos Lampreave, Alberto Cebollada, Isabel Otal, Sofía Samper, Carlos Martín

**Affiliations:** 1 Grupo de Genética de Micobacterias, Departamento de Microbiología y Medicina Preventiva. Facultad de Medicina, Universidad de Zaragoza, IIS Aragón, Zaragoza, Spain; 2 CIBER Enfermedades Respiratorias, Instituto de Salud Carlos III, Madrid, Spain; 3 Instituto de Biocomputación y Física de Sistemas Complejos (BIFI), Zaragoza, Spain; 4 Unidad de Investigación Translacional, Hospital Universitario Miguel Servet, Instituto de Investigación Sanitaria Aragón. Zaragoza, Spain; 5 Servicio de Microbiología, Hospital Universitario Miguel Servet, Zaragoza, Spain; Institut Pasteur, CNRS UMR 3525, FRANCE

## Abstract

The insertion Sequence IS*6110*, only present in the pathogens of the *Mycobacterium tuberculosis* Complex (MTBC), has been the gold-standard epidemiological marker for TB for more than 25 years, but biological implications of IS*6110* transposition during MTBC adaptation to humans remain elusive. By studying 2,236 clinical isolates typed by IS*6110*-RFLP and covering the MTBC, we remarked a lineage-specific content of IS*6110* being higher in modern globally distributed strains. Once observed the IS*6110* distribution in the MTBC, we selected representative isolates and found a correlation between the normalized expression of IS*6110* and its abundance in MTBC chromosomes. We also studied the molecular regulation of IS*6110* transposition and we found a synergistic action of two post-transcriptional mechanisms: a -1 ribosomal frameshift and a RNA pseudoknot which interferes translation. The construction of a transcriptionally active transposase resulted in 20-fold increase of the transposition frequency. Finally, we examined transposition in *M*. *bovis* and *M*. *tuberculosis* during laboratory starvation and in a mouse infection model of TB. Our results shown a higher transposition in *M*. *tuberculosis*, that preferably happens during TB infection in mice and after one year of laboratory culture, suggesting that IS*6110* transposition is dynamically adapted to the host and to adverse growth conditions.

## Author summary

Since the pioneering discovery of transposition by Barbara McClintock in eukaryotes and later in prokaryotes by Robert W. Hedges and Alan E. Jacob, it has become clear the key role of mobile genetics elements in chromosome remodelling, microbial evolution and host adaptation. The insertion sequence IS*6110* is widely recognized for its utility in TB diagnosis and epidemiology because it is only present in the *M*. *tuberculosis* Complex (MTBC) and its transposition provides an excellent chromosomal polymorphic variability allowing the study of recent TB transmission. This inherent feature of IS*6110* leads us to hypothesize that IS*6110* plays a crucial role during the TB infectious cycle. However, the biological significance of IS*6110* has been hindered by its almost exclusive use as an epidemiological marker. Here, we study the regulatory mechanisms and the distribution of IS*6110* in the different MTBC lineages. We discuss the potential biological implications of IS*6110*, that is much more than an excellent TB epidemiological tool. Since IS*6110* could play an important role in the adaptation of MTBC to the host, this study opens new avenues to decipher the biological roles of IS*6110* in TB pathogenesis.

## Introduction

Tuberculosis (TB) is the largest infectious cause of death in history having claimed more deaths than smallpox, malaria, plague, influenza and AIDS together [1]. In addition to the alarming 1.7 million deaths and 10,4 million of new TB cases in 2016, the emergence of multi-drug resistant strains is an increasing threat which makes TB treatment difficult or occasionally impossible [2]. Thus, early diagnostics and identification of transmission chains greatly contribute to control the TB epidemic.

The adaptation of *M*. *tuberculosis* to the host is extremely complex. Most of the infected individuals are chronically infected in the form of latent TB infection (LTBI) and only one of 10 will develop clinical TB disease. The essential, yet unanswered question, on the natural history of TB is when *M*. *tuberculosis* decides to establish either LTBI in the host, resembling the lysogenic cycle of lambda phage, or to cause pulmonary TB disease, like the lytic cycle of lambda phage. In this latter case, *M*. *tuberculosis* decide to kill the host with the aim of achieving transmission to new hosts [3].

Seminal studies by Barbara McClintock deciphered the key role of mobile genetic elements in chromosome remodelling of maize in 1950 [4]. In the late 60’s insertion sequences were described by the groups of Shapiro, Malamy, Sybalsky and Starlinger and in 1974 Robert W. Hedges and Alan E. Jacob coined the term “transposition” in bacteria [5]. The insertion sequence IS*6110* is a mobile genetic element exclusively found in the *M*. *tuberculosis* Complex (MTBC) [6], the causative agent of TB in humans and other mammals including farm animals responsible for zoonotic TB transmission. This feature makes IS*6110* a valuable tool in the diagnosis of MTBC in biological samples [7, 8]. In addition, IS*6110* is present in multiple copies in the chromosome of *M*. *tuberculosis* and IS*6110* restriction fragment length polymorphism (RFLP) analysis of strains isolated from patients who developed TB showed identical patterns over years [9]. On the other side a high degree of polymorphism was observed between strains of the MTBC isolated from different patients due to IS*6110* transposition [10]. Standardized IS*6110* RFLP typing has been the gold standard for more than 25 years, being the most reliable TB epidemiological marker [11]. IS*6110* typing allows the detection of TB outbreaks as well as to identify transmission chains using conventional and molecular methods [12]. To date tens of thousands of MTBC stains all around the world have been typed by this method but the biological role, if any, of IS*6110* remains elusive. In the last 5–10 years IS*6110* typing is being replaced by less time-consuming methods based in PCR amplification of mycobacterial interspersed repetitive units (MIRU) [13, 14], or more recently by whole genome sequencing (WGS) [15, 16].

The MTBC comprises eight defined phylogenetic lineages. *M*. *tuberculosis sensu*-*stricto* includes lineages L1–L4 and L7. These human-adapted lineages are responsible for the vast majority of global human TB cases, whereas *M*. *africanum* lineages (L5, L6) are mainly restricted to humans from West Africa. The L8 comprises animal-adapted strains with ecotypes adapted to different mammals, such as *M*. *caprae* and *M*. *bovis*, which branched from the *M*. *africanum* lineage [17]. All these lineages are classified into sub-lineage / clonal complexes or families on the basis of different spoligotyping profiles [18] or on specific genomic signatures [19, 20]. The more distantly related *M*. *canettii* is outside the clonal population of the MTBC and it is considered the most ancestral progenitor from which the above mentioned MTBC members emerged [21].

According to the IS*6110* content, MTBC members are classified into high (>6) and low (<7) IS*6110* copy number strains [22]. It is not clear whether differences in IS*6110* content account for biological phenotypic consequences in bacterial physiology and pathogenesis. However, it is well known that the *M*. *tuberculosis* Beijing/W lineage (L2), with a remarkably high content of IS*6110* [23], is associated with higher virulence and massive spread of drug resistant strains, being possibly better adapted to high density populations [19]. Beijing/W lineage was originally described in the 1990’s as a predominant genotype found in countries of East Asia designated Beijing-family [24] and after observing an interstate spread from New York of the multidrug-resistant *M*. *tuberculosis* clone family named W [25].

During its transposition, the IS*6110* promotes a number of important genetic modifications in MTBC strains. This confers plasticity to the MTBC genomes and could have significant biological implications. As for other IS, insertion of IS*6110* into a coding region frequently renders the gene inactive, the basis of transposon mutagenesis, or the recombination between two IS*6110* copies can lead to either inversion or deletion of the chromosomal fragment between them [26–28]. Furthermore, it has been demonstrated that IS*6110* acts as a mobile promoter and this phenotype is selectively activated during *in vitro* infection of monocytes/macrophages [29, 30]. This latter finding has extraordinary consequences in the host-pathogen evolution of the MTBC, as will be discussed below.

It has been suggested that a moderate number of IS*6110* might translate into strain-specific phenotypes that provide selective advantages during the course of the infection [26]. Conversely, it has been demonstrated that excessive accumulation of IS*6110* copies could result in inactivation or deletion of essential genetic regions, being deleterious to the bacterium [31]. This later finding implies that transposition rates of IS*6110* should be finely regulated and maintained at relatively low levels (7.9x10^-5^ events per site per generation) [32]. Considering the clonal evolution of the MTBC, the rate of point mutations is estimated at 10^−9^ events per site per generation and comparatively the mutation rate of IS*6110* is orders of magnitude higher. This reinforces the notion that IS*6110* transposition is under positive selection when infecting or causing disease to the host [32] and accordingly it constitutes an excellent TB epidemiological marker.

At the genetic level the IS*6110* belongs to the IS*3* family and it is annotated as two open reading frames: ORF1 (327 bp) and ORF2 (987 bp) which overlap in 52 bp and are flanked by 28 bp imperfect Inverted Repeats (IR). The 3–4 bp boundaries of IS*6110* are duplicated upon transposition [10]. Despite the massive use of WGS, the repetitive nature of IS*6110* makes difficult to finely map their localizations in the MTBC chromosomes. Although some studies have attempted to localize IS*6110* in *M*. *tuberculosis* genomes [27, 33–35], little is known about its involvement in other MTBC members including *M*. *canettii*, *M*. *africanum* and ecotypes responsible for animal TB (i.e. *M*. *bovis* and *M*. *caprae*) which possess a zoonotic risk.

Similar to other members of the IS*3* family, it is thought that transposition of IS*6110* occurs when its two constituent ORFs are translationally fused producing an active transposase [36]. Former studies using *M*. *smegmatis* (a non-pathogen fast growing mycobacteria) as surrogate host to demonstrate that IS*6110* transposition occurs more readily when this element is located in transcriptionally active locations and also upon exposure to a microaerobic environment [37, 38]. However, there is a definite lack of evidence about the precise mechanisms leading to the production of an active IS*6110* transposase and the physiological conditions that promote transposition in the MTBC. In the present study, we analyse biological data from more than two-thousand clinical isolates covering the MTBC to dissect the molecular mechanism of IS*6110* transposition and its dynamic distribution between the different MTBC lineages. We discuss its biological significance in the tubercle bacillus and also in the clinical presentations of TB.

## Results

### IS*6110* copy number in the MTBC is lineage-specific

**Fig 1:**
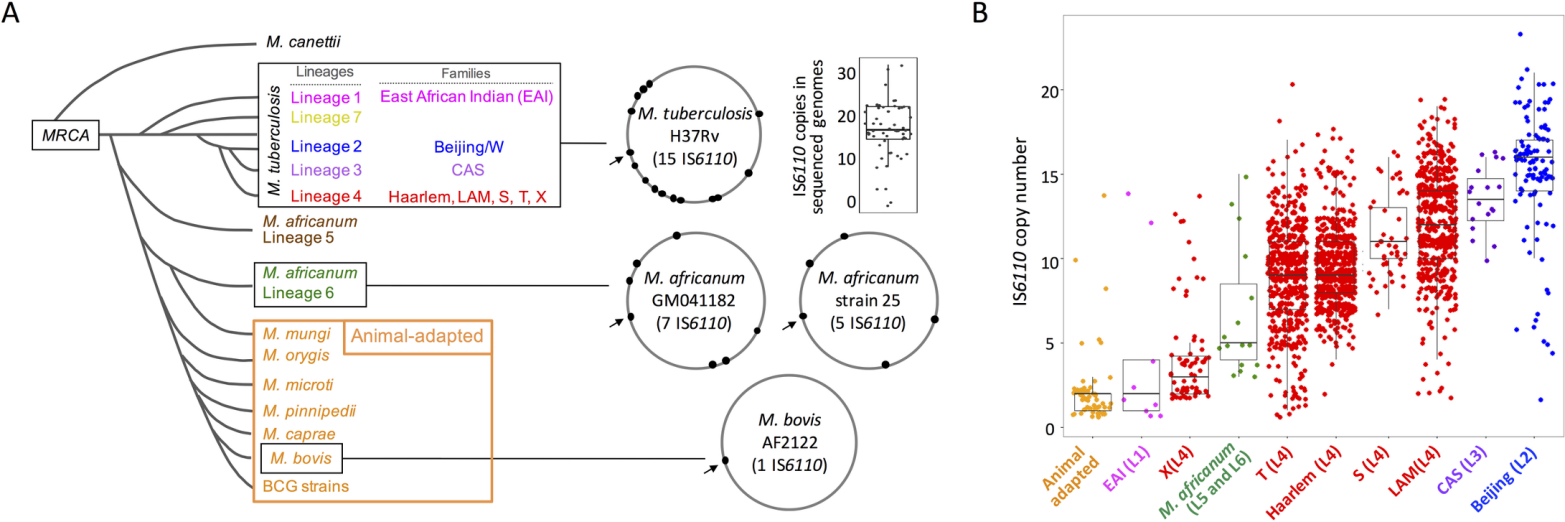
IS*6110* in the MTBC. (**a**) Schematic phylogenetic relationships of MTBC members arisen from a most recent common ancestor (MRCA) after an evolutionary bottleneck. For *M*. *tuberculosis* different lineages and families are indicated. The position of IS*6110* sequences in fully assembled genomes in indicated by black dots. The arrow indicates the position of IS*6110* in the Direct Repeat region of the CRISPR-Cas locus, which is common to most MTBC strains. For the remaining 17 *M*. *tuberculosis* strains different from H37Rv, the number of IS*6110* sequences is indicated by a box plot (median = 17). (**b**) Box plots showing the IS*6110* copies in MTBC families. For each family, the lineage according to panel (a) is provided in parenthesis in the X-axis. For clarity, L4 have been subdivided into 5 different families according to spoligotyping.

Different members from the MTBC have evolved by accumulation of genomic deletions and specific polymorphisms [39, 40]. Accordingly, the MTBC phylogeny is the result of a genomic decay after an evolutionary bottleneck which led to speciation [41]. Upon examination of fully sequenced and assembled MTBC genomes, we observe that the *M*. *bovis* AF2122/97 reference strain contains a single IS*6110* while *M*. *africanum* and *M*. *tuberculosis* have higher copy numbers of this element (an average of 6 and 17 respectively) (**Fig 1A**). When interrogating *M*. *canettii*, considered as the most ancestral linage known from which all MTBC members emerged, we only found potentially functional IS*6110* sequences in subgroups STB-A, -D, and–L. Those subgroups that show greater phylogenetic distances (STB–J and–K) have no traces of IS*6110* [21]. Only STB-L carries identical IS*6110* sequences to the MTBC (**S1 Fig**). Supporting this finding, another study demonstrated the presence of IS*6110* in evolutionarily closer *M*. *canettii* isolates [42].

The IS*6110* content in MTBC genomes suggested to us that the copy number of this transposon might be lineage-specific. However, since a limited number of genomes have been fully sequenced and assembled, we decided to investigate this hypothesis in a representative collection of TB causing strains. We systematically genotyped clinical isolates from TB patients during the last 25 years and subdivided them in families according to spoligotyping profiles. A total of 2,236 clinical samples from our data base covering the MTBC were analysed by standardised Restriction Fragment Length Polymorphism (RFLP) of IS*6110*. Results confirmed that the average IS*6110* content is lineage-specific, ranging from low copy number (*M*. *bovis*, L1, the L4 sub-lineage X and *M*. *africanum* L5 and L6) to high copy number in modern *M*. *tuberculosis* lineages (LAM, CAS and Beijing from L4, L3 and L2 respectively) (**Fig 1B)**. Among the *M*. *tuberculosis* human-adapted species, these high copy number families are globally distributed and accordingly they could be considered as generalists capable of infecting and causing disease in many different human populations [43]. Of these, the LAM family is distributed in America, Africa, Europe, Oceania and East Asia [20], the CAS family affects the Indian continent and East Africa [44] and the Beijing family is amply distributed in East Asia and East Europe [19].

### Normalised IS*6110* expression data indicates an exponential transposition dynamics in the MTBC

**Fig 2:**
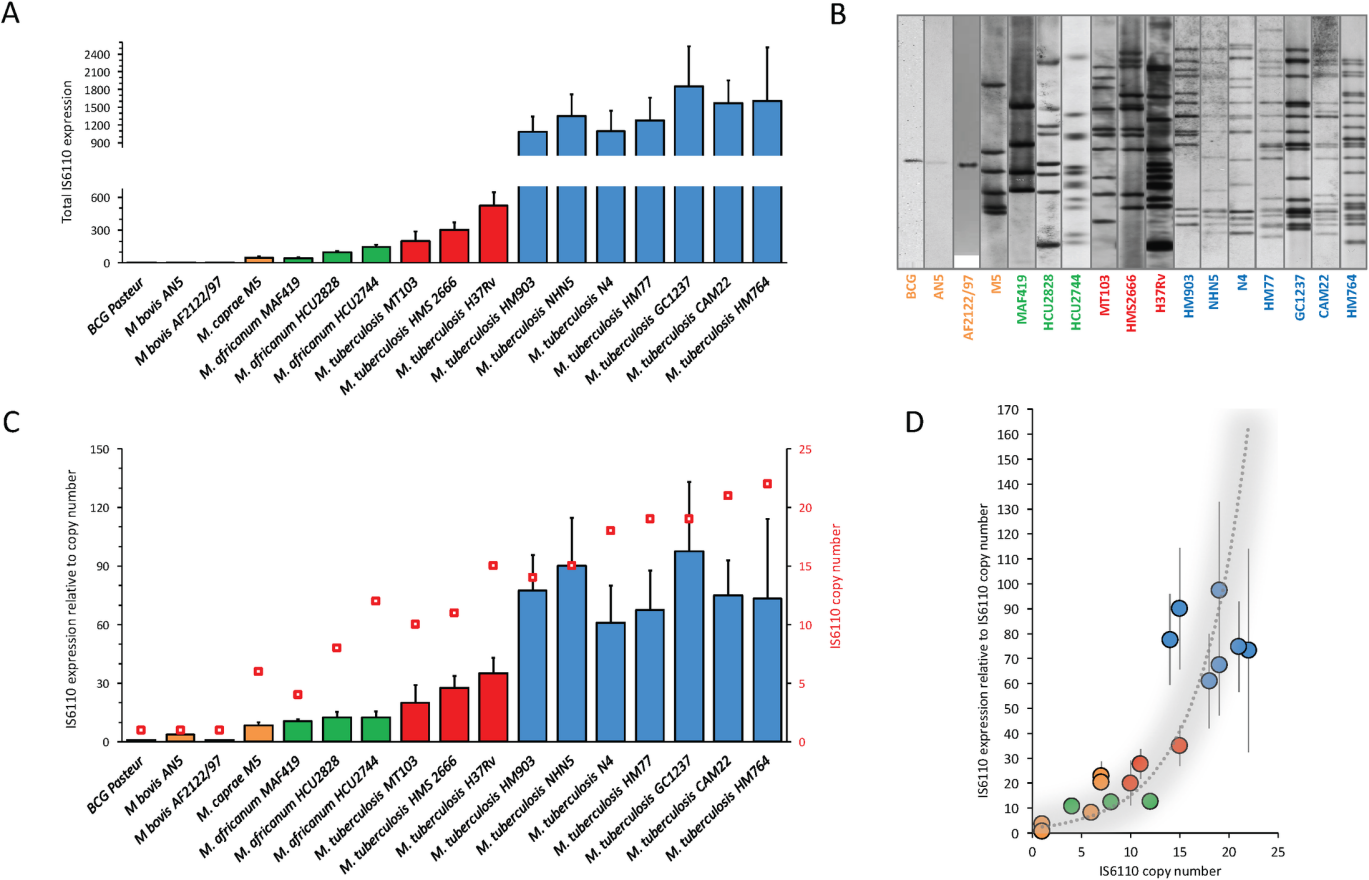
IS*6110* gene expression and determination of transposition dynamics in the MTBC. (**a**) Total IS*6110* expression in representative strains from the MTBC. Data are relative to BCG Pasteur. Columns and error bars are the average and standard deviation from three independent cultures. (**b**) IS*6110* RFLP from MTBC strains analysed in panel (a). (**c**) IS*6110* expression values normalised to the copy number content of this element. Columns represent normalised expression of IS*6110* according to the left Y-axis. Red squares show the IS*6110* copy number in each strain indicated in the right Y-axis. Normalised expression of BCG Pasteur is used as reference. (**d**) Expression per *IS6110* copy relative to the copy number content in MTBC strains. Data fit with an exponential curve (r^2^ = 0.80) indicated by a grey shadowed line.

Once we established that MTBC phylogenetic clades have different IS*6110* content, we interrogated the molecular mechanisms underlying this observation. First, we selected representative isolates of the MTBC (BCG, *M*. *bovis*, *M*. *caprae*, *M*. *africanum* and *M*. *tuberculosis*). Then we analysed their global IS*6110* mRNA levels and found that animal-adapted and *M*. *africanum* species have lower levels of IS*6110* mRNA than *M*. *tuberculosis* L2 and L4 (**Fig 2A**). We also found that ORF1 and ORF2 were similarly expressed (**S2 Fig**), with is compatible with the presence of a single RNA molecule with two out-of-phase reading frames translated into a single ORF by way of a translational frameshift. This result resembles other IS3 family members [36, 45]. Altogether these results indicated a proportional relationship between the copy number content and IS*6110* mRNA expression, which led us to quantitate the “normalised mRNA expression” by calculating expression ratios relative to the IS*6110* content in every MTBC strain (IS*6110* mRNA / IS*6110* copy number). First, the IS*6110* copy number in the above-mentioned strains was checked by RFLP (**Fig 2B**) and our previous results were reanalysed considering this IS*6110* content. Our results demonstrated that expression per IS*6110* copy is lower in animal adapted strains and in *M*. *africanum* than in *M*. *tuberculosis* (**Fig 2C**).

IS*6110* distribution in representative MTBC members indicates a proportional relationship between the copy number content and the normalised expression of this element and this relation follows an exponential trend (r^2^ = 0.80) (**Fig 2D**). To gain further insight into the transposition dynamics of IS*6110*, we analysed *M*. *bovis* and *M*. *tuberculosis* isolates showing an uncommon copy number of this element. We selected *M*. *tuberculosis* clinical isolates from X and T families of lineage 4 containing 1, 2, 3 and 11 IS*6110* copies. Beijing strains from lineage 2 known to possess the highest copy number of IS*6110* were also included [34]. We also selected *M*. *bovis* strains causative of human TB with 3, 4 and 5 copies of *IS6110* [46], which represent an unusually high copy number for the animal adapted lineage (**S3 Fig**). Results demonstrated that *M*. *tuberculosis* strains having a single IS*6110* expresses this mRNA similarly to *M*. *bovis* BCG. Accumulation of additional copies of IS*6110* resulted in higher mRNA expression of the transposase to reach expression levels comparable to *M*. *tuberculosis* H37Rv (**S3 Fig**). On the other hand, accumulation of more than one IS*6110* in *M*. *bovis* resulted in exacerbated expression of its coding gene. This expression was 5-fold higher than that observed in *M*. *tuberculosis* H37Rv even if the latter contains 15 IS*6110* compared to the 3–5 copies in these atypical *M*. *bovis* isolates (**S3 Fig**).

### Translation of an active IS*6110* transposase is post-transcriptionally regulated by a ribosomal frameshift and a RNA pseudoknot

**Fig 3:**
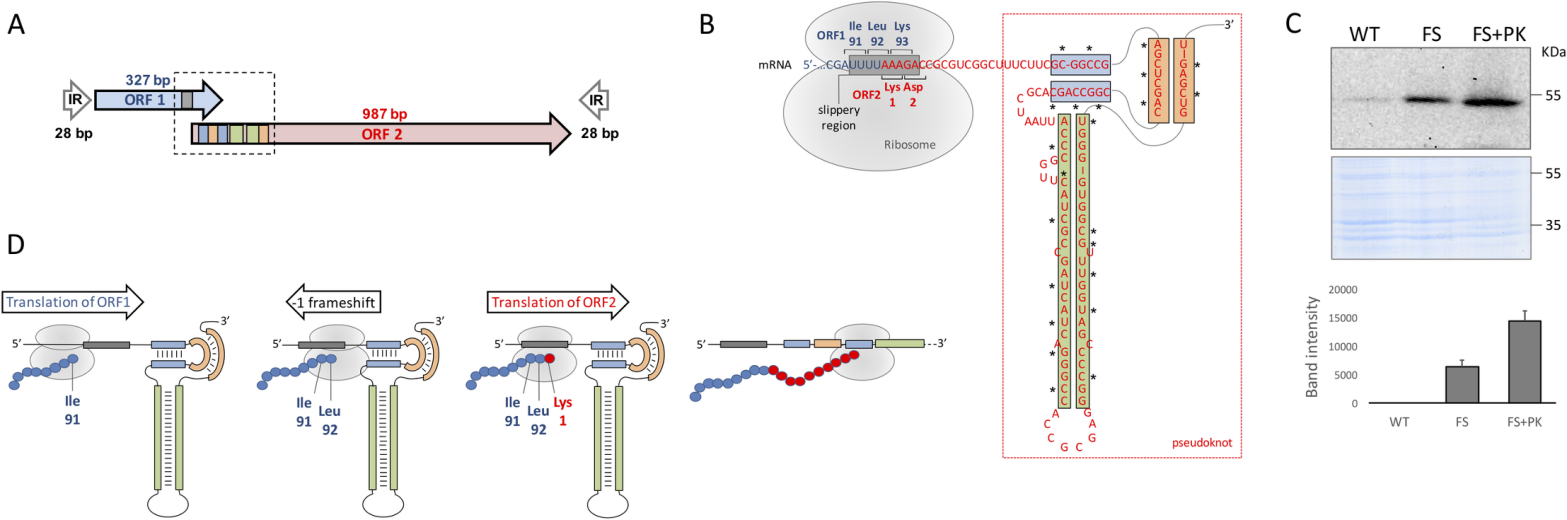
Post-transcriptional regulatory mechanisms of IS*6110* translation. (**a**) Genetic organization of IS*6110*. Overlapping ORF1 and ORF2 and the sense of transcription are indicated as blue and red arrows respectively. The scheme also shows the 28bp inverted repeats (IR) flanking both overlapping ORFs. (**b**) Mechanisms of post-transcriptional regulation of IS*6110*. The image shows an enlarged view of the region indicated with a dotted box from panel (a). The UUUUAAAG slippery sequence is indicated by a grey box. ORF1 and ORF2 as well as their coding triplets are indicated by blue and red letters according to panel (a). The RNA pseudoknot is included within a red rectangle and those regions involved in base pairing formation of secondary structures are indicated by blue, green and orange boxes. The position of the ribosome and the translated codons are also indicated. Asterisks in the pseudoknot indicate positions carrying mutations that disrupt this structure. (**c**) Expression of 3xFLAG variants of IS*6110*-WT, the transcriptionally active transposase IS*6110*-FS and the latter variant carrying mutations to disrupt pseudoknot formation IS*6110*-FS+PK. The upper and lower parts of the panel show a western-blot using and anti-FLAG antibody and a Coomassie staining which serves as loading control respectively. The right side of the panel shows the band intensity average from three independent experiments. (**d**) Post-transcriptional regulation of IS*6110* to produce a biologically active transposase. The image shows translation steps indicating the sense of ribosomal advance and the mRNA structure indicated in panel (b). Translation of the ORF1 produces the aminoacids from the N-terminus of IS*6110* (blue spheres) until it translates Ile91 and Leu92 coded by AUU and UUA triplets in the slippery region (grey box). At this position ribosome stalls probably because the presence of the downstream pseudoknot presenting a tight secondary structure. Stalling favours a -1 frameshift in the slippery region. Translation continues in the AAA codon coding for the Lys1 position of ORF2 (red sphere) until the ribosome reaches the C-terminus of IS*6110* coded in this latter ORF.

The use of IS*6110* as molecular epidemiological marker is useful due to its relatively low frequency of transposition which allows investigators to distinguish between currently circulating strains (transmission) and older episodes of TB (reactivation) in individual patients. Since transcription per IS*6110* copy is within the range of other genes producing physiological phenotypes in *M*. *tuberculosis* (**S4 Fig**), it is predictable that low transposition rates must be subjected to some type of post-transcriptional regulation. Our results show that both ORFs are similarly transcribed (**S2 Fig**). The transposase is composed of a DNA binding domain (N-term) and a catalytic integrase domain (C-term) which contains the residues forming the putative active site (D310, D350, E379) (**S5A and S5B Fig**). By analysing the IS*6110* genetic sequence we found that the intergenic region of the constituent ORFs contained a putative translational frameshift that could produce an active transposase as described for other members of the IS*3* family [47]. Since the precise translational frameshift has not been documented for IS*6110*, we searched for heptanucleotide U/A-rich sequences defined by the motif XXX-YYY-Z [47] in the overlapping region of ORF1 and ORF2 since these sequences are prone to ribosomal slippage. A auUUU-AAA-Gac sequence was located in the appropriate location (**Fig 3A and 3B**). This sequence codes for Ile91 (AUU), Leu92 (UUA) and Lys93 (AAG) codons of ORF1 and upon translational slippage it codes for Lys1 (AAA) and Asp2 (GAC) of ORF2 (**Fig 3B**). Additionally, we found a tight RNA secondary structure known as pseudoknot immediately downstream of the slippage sequence (**Fig 3A and 3B**). Pseudoknots are very complex and stable RNA structures with diverse biological functions, which include self-catalytic activity or the induction of ribosomal frameshifting [48]

To validate these mechanisms, we constructed three genetic variants of IS*6110* fused to a 3xFLAG epitope in order to detect the functional transposase by western blot. These variants were: the wild type (WT) sequence containing the UUU-AAA-G slippage region (IS*6110*-WT-FLAG), a construct with an A insertion in the slippage sequence (UUU-AAAA-G) to produce a complete transposase in the absence of ribosomal frameshift (IS*6110*-FS-FLAG) and a third construct including the previous A insertion and several mutations to disrupt pseudoknot formation without affecting the coding sequence (IS*6110*-FS+PK-FLAG) (**S6 Fig**). These variants were introduced in *Escherichia coli* to detect IS*6110* protein expression. We barely detected the IS*6110* using the WT sequence. In contrast, by introducing an A insertion a transcriptionally active transposase was detected as a discrete band (**Fig 3C**). Further, introduction of mutations in the pseudoknot sequence resulted in even more increased translation of the functional transposase (**Fig 3C**). Based on these findings, we infer that post-transcriptional regulation of IS*6110* occurs by the combined action of two genetic mechanisms inherent to its coding sequence. The presence of a slippage sequence and a downstream pseudoknot would favour ribosome stalling at the appropriate location and the subsequent -1 translational frameshift (**Fig 3D**). In addition, the 5’ end of the IS*6110* transcript is predicted to form a hairpin structure which occludes the ribosome binding sequence (**S5C Fig**) and possibly interferes with translation.

### Transcriptionally active IS*6110* results in higher transposition frequencies during laboratory growth

**Fig 4:**
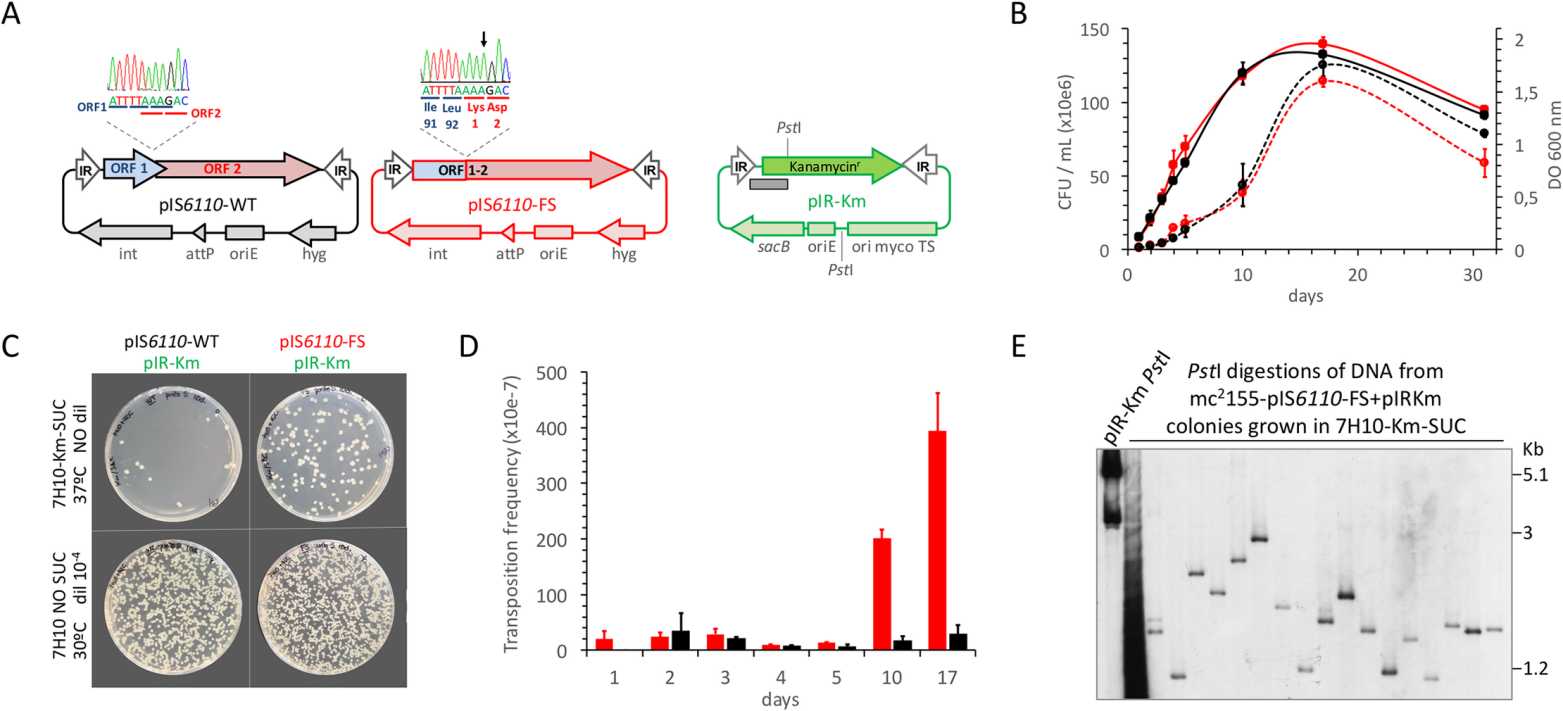
Construction of a transcriptionally active IS*6110* and measure of transposition frequencies during laboratory growth. (**a**) Plasmids used in the transposition reporter system. pIS*6110*-WT and pIS*6110*-FS are mycobacterial integrative plasmids carrying either the wild type IS*6110* or a mutated variant producing a transcriptionally active transposase respectively. The upper side of this panel shows Sanger sequencing histograms indicating the position of the A insertion in pIS*6110*-FS. pIR-Km is a conditionally replicating plasmid with thermosensitive origin of replication and the *sacB* gene conferring sucrose sensitivity. This plasmid contains a Kanamycin resistance cassette (km) flanked by the IR regions of IS*6110*. Positions of *Pst*I sites and probe (grey rectangle) used in RFLP shown in panel (e) are indicated. (**b**) Growth rates of liquid cultures at 30°C of *M*. *smegmatis* transformed with either pIS*6110*-WT+pIR-Km (black lines) or pIS*6110*-FS+pIR-Km (red lines). Growth curves measured by OD_600_ and enumeration of CFU/mL are represented by continuous or dotted lines respectively. Error bars represent the standard deviation from three independent cultures. (**c**) CFU from *M*. *smegmatis* cotransformed with pIS*6110*-WT+pIR-Km or pIS*6110*-FS+pIR-Km and plated on 7H10 media supplemented with or without kanamycin and sucrose. Dilution used and incubation temperature are indicated. Note the increase in the number of CFU grown in kanamycin and sucrose medium for the pIS*6110*-FS variant relative to the pIS*6110*-WT. (**d**) Determination of transposition frequencies in *M*. *smegmatis* cotransformed with pIS*6110*-WT+pIR-Km (black columns) or pIS*6110*-FS+pIR-Km (red columns). Error bars indicate the standard deviation from three independent experiments. Note that the transcriptionally active transposase in pIS*6110*-FS increases up to 20-fold its transposition frequency relative to the wild type transposase in exponential and stationary periods. (**e**) RFLP analysis of DNA from colonies grown in kanamycin and sucrose plates resulting from transposition events. Note the loss of signal for pIR-Km indicative of the appropriate plasmid loss and the concomitant presence of an aleatory band pattern indicative of randomised transposition in the *M*. *smegmatis* chromosome.

Our next step was to demonstrate that the IS*6110* transposase produced after translational frameshift is biologically active when mycobacteria are grown under laboratory conditions. To avoid homologous recombination or other potential confusing effects that could be produced from orthologue IS*6110* sequences, we decided to study transposition in *M*. *smegmatis* mc^2^155, a fast growing, non pathogen mycobacterial surrogate host in which the IS*6110* is not present [49]. It is important to remark that IS*6110* is exclusive of the MTBC and albeit a related IS*6110* (67% aminoacid identity) has been found in the MKD8 strain of *M*. *smegmatis*, this copy is non functional [49]. We cloned in a mycobacterial integrative plasmid either the wild type (pIS*6110*-WT) or a variant carrying the A insertion in the slippage region (pIS*6110*-FS) expected to be transcriptionally active (**Fig 4A**). Active transposases recognize the ends flanking the transposon, which in the case of IS*6110* are the IR, and catalyse “copy-out-paste-in” transposition [50, 51]. Accordingly, we constructed a third plasmid to act as a transposition reporter. A kanamycin resistance cassette flanked by the IR regions of the IS*6110* was cloned in a conditionally replicating plasmid with thermosensitive origin and *sacB* counter-selectable marker and named pIR-Km (**Fig 4A**). Plasmid pIR-Km was introduced in *M*. *smegmatis* mc^2^155 carrying either pIS*6110*-WT or pIS*6110*-FS and maintained at 30°C. We confirmed that both strains grew at comparable rates (**Fig 4B**). To measure transposition frequency, aliquots were plated on 7H10 medium to enumerate total CFU or on 7H10 medium containing kanamycin and sucrose and incubated at 42°C. Under these latter conditions pIR-Km does not replicate and consequently kanamycin and sucrose resistant colonies arise from transposition of the IR-Km-IR construct into the chromosome (**Fig 4C**). Our results revealed that the transcriptionally active transposase in pIS*6110*-FS exhibited 20-fold higher transposition rates than the wild type IS*6110* (**Fig 4D**). Differences in transposition frequencies between both transposase variants were notably significant during the exponential and early stationary growth with a higher proportion of colonies resulting from transposition in this latter phase (**Fig 4D**). This result opens the door to hypothesize whether transposition *in vitro* is phase-dependent or conversely it results from accumulation of transposition events during mycobacterial growth. In order to confirm that transposition occurs randomly across *M*. *smegmatis* chromosome, we used a similar RFLP-IS*6110* analysis to that used in MTBC clinical isolates. Several kanamycin and sucrose resistant colonies were chosen at random and their restriction fragments were hybridised with a probe against the IR-Km-IR fragment. The RFLP showed loss of signal from the pIR-Km indicative of the appropriate plasmid loss. A polymorphic RFLP pattern was observed, indicative that IS*6110* transposition occurred at random locations in the chromosome (**Fig 4E**).

### Transposition in *M*. *bovis* and *M*. *tuberculosis* preferentially occurs during laboratory starvation and in a mouse infection model of TB

**Fig 5:**
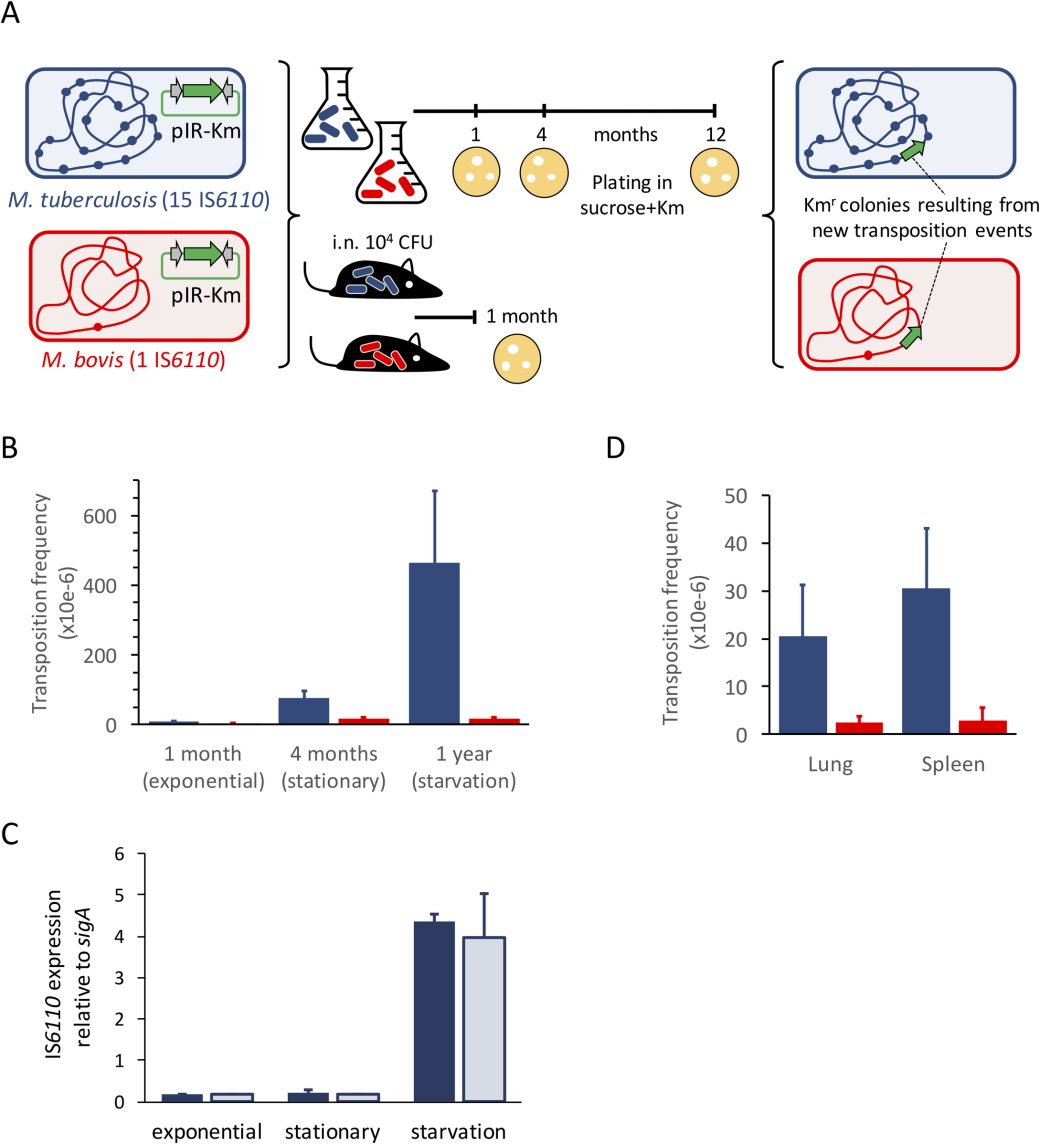
Transposition in the laboratory and in a mouse infection model using reference *M*. *bovis* and *M*. *tuberculosis* strains. (**a**) Experimental model to measure transposition rates in *M*. *tuberculosis* H37Rv (15 IS*6110* copies) and *M*. *bovis* AF2122/97 (1 IS*6110* copy). Both strains are transformed with pIR-Km and used to inoculate liquid media or to intranasally infect C57BL/6 mice. After the indicated time points aliquots are plated in kanamycin and sucrose containing plates to ensure pIR-km loss and to recover colonies resulting from transposition. (**b**) Transposition frequencies in laboratory medium in *M*. *bovis* and *M*. *tuberculosis* are indicated by red and blue columns respectively. Error bars indicate the standard deviation of the mean value from three independent cultures. Transposition preferentially occurs in *M*. *tuberculosis* after the stationary phase reaching it maximum in the starvation period. (**c**) Expression of *M*. *tuberculosis* IS*6110* during exponential, stationary and starvation periods *in vitro*. Expression of ORF1 and ORF2 are indicated by dark and light blue columns respectively. Results are from three independent cultures. (**d**) Transposition frequencies during mouse infection with *M*. *bovis* or *M*. *tuberculosis* are indicated by red and blue columns respectively. Data from lung and spleen are shown and error bars indicate the standard deviation of the mean value from three independent mice. *M*. *bovis* does not exhibit increased transposition rates *in vivo* relative to liquid culture. Conversely *M*. *tuberculosis* show 10-fold higher transposition rates compared to exponential growth *in vitro*. In all cases, transposition frequencies were calculated relative to the total number of CFU in either cultures or mouse organs.

Once studied the IS*6110* distribution in more than 2.000 strains covering various MTBC lineages and after we have experimentally demonstrated that low transposition frequencies of IS*6110* are due to a post-transcriptional mechanism in *M*. *smegmatis*, we pursued our investigations in analysing potential biological conditions” promoting IS*6110* transposition in slow growing MTBC. We chose as reference strains *M*. *tuberculosis* H37Rv (15 IS*6110* copies) belonging to L4 and *M*. *bovis* AF2122/97 (1 IS*6110* copy) as representative of the animal-adapted L8. Each strain was transformed with the IS*6110* transposition reporter pIR-Km plasmid to measure transposition during growth on laboratory media or in a mouse infection model of TB. Aliquots of the culture or from organ homogenates at different time points were plated on conventional 7H10 medium to enumerate total CFU or on 7H10 supplemented with sucrose and kanamycin to recover colonies resulting from IS*6110* transposition (**Fig 5A**). For *in vitro* transposition experiments, we first confirmed that both strains carrying pIR-Km grew at comparable rates at 30°C, a permissive temperature for this plasmid (**S7 Fig**). Then, we selected 1, 4 and 12 months’ time points as representative for exponential, stationary and starvation periods in *in vitro* cultures according to growth curves at 30°C (**S7 Fig**). Our results for *M*. *tuberculosis* H37Rv indicate that transposition rates were 10- and 60-fold higher in stationary and starvation periods respectively relative to exponential growth (**Fig 5B**). When examining *M*. *bovis* AF2122/97, similar transposition frequencies were observed under exponential growth with respect to *M*. *tuberculosis*. However, although transposition in *M*. *bovis* strain was 5-fold higher in stationary and starvation periods, this was noticeably lower than that observed for *M*. *tuberculosis* (**Fig 5B**). We also quantitated *M*. *tuberculosis* IS*6110* expression during laboratory growth and we found higher mRNA transcription in the starvation period (**Fig 5C**). This result indicates that even if high mRNA expression does not necessarily imply high translation rates, there is a remarkable correlation between the transposase expression and the transposition frequencies. These results indicate that transposition increases starting from the stationary growth and similarly to that observed in *M*. *smegmatis* we cannot rule out the possibility that transposition events accumulate during growth *in vitro*. Further, the comparison of both strains allows us to establish lineage-defined transposition frequency. These results are remarkably comparable with our previous findings indicating that normalised expression of IS*6110* is lineage-specific, being 35-fold higher in *M*. *tuberculosis* than in *M*. *bovis* (**Fig 2C**).

Our transposition experiments in mice correlate with our findings during laboratory growth with an interesting exception: transposition rates for *M*. *bovis* AF2122/97 did not differ in the mouse model relative to exponential growth in laboratory medium, both being in the order 10^−6^ (**Fig 5B and 5D**). This result agrees with current biological and clinical data indicating that the single IS*6110* copy of *M*. *bovis* strains has been maintained during evolution with rare cases of transposition in this lineage. Further supporting these observations, our previous results demonstrate marginal levels of normalised IS*6110* mRNA expression in *M*. *bovis* isolates (**Fig 2C**). Conversely, for *M*. *tuberculosis* H37Rv, we observed a 10-fold increase in the transposition rates during mouse infection relative to exponential growth (**Fig 5D**). This increase was observed not only in the lung -the primary site of infection-, but also in the spleen of infected animals (**Fig 5D**). Finally, we also demonstrate that normalised expression of IS*6110* increases upon infection of murine alveolar macrophages (**S8 Fig**) and this result supports our transposition experiments in the mouse model of TB.

## Discussion

The IS*6110* belongs to the IS*3* family whose more representative member is IS*911*. In this work, we first demonstrate that similarly to IS*911*, the IS*6110* is subjected to -1 ribosomal frameshifting [36, 52], contains a RNA pseudoknot [47] and its transposition occurs by a copy-out-paste-in mechanism [53–55]. Then, we go a step ahead to understand the biological role of IS*6110* transposition in the MTBC biology. The reliability of IS*6110* as a clinical epidemiological marker is unquestionable. In 1993 DNA fingerprinting using IS*6110* was standardized and became the gold standard for epidemiological studies of TB in the last 25 years. Since then tens of thousands MTBC strains have been studied by IS*6110* RFLP [11]. IS*6110* RFLP requires extraction of DNA from pure cultures/sputum samples which is then used in a Southern-Blot hybridization. Consequently, this is a laborious and time-consuming technique that in the last 5–10 years is being replaced by PCR methods based on amplification of MIRU [13, 14] or even more recently, by WGS [15, 16]. However, MIRU does not allow to know the number and position of IS*6110* insertions in the MTBC strains and most WGS studies fail to determine the number and localizing repeated sequences in the genome, such as the insertion sites of IS*6110*. Hopefully, new PacBio and Oxford Nanopore sequencing technologies will improve the resolution of WGS.

After an in depth systematic analysis of 2,236 clinical isolates typed by IS*6110*-RFLP our findings show the different distribution of IS*6110* between the various MTBC lineages. Our results reveal that modern lineages of the MTBC (L2, L3 and L4) have accumulated higher IS*6110* copy number than ancient lineages (L1, L5 and L6) (**Fig 1)**. Since modern lineages are widely distributed and consequently they are more successfully adapted to high density populations they have been referred to as generalists [43]. Conversely, lineages geographically restricted to certain regions are considered specialists [43]. Given the role of mobile genetic elements in providing chromosomal variability, it is tempting to think that the higher IS*6110* number in generalists might represent a strategy of the MTBC to adapt to different populations. A potential limitation of our study is the predominance of strains corresponding to L4, more frequent in Europe, Africa and America. Similar studies in other places of the world using larger number of the remaining MTBC lineages would be important to confirm the results of the present study.

As with any mutational event, transposition could be deleterious, neutral or advantageous and these events might impact on the pathogen fitness. Accordingly, another limitation of our study is inherent to the use of clinical isolates since only advantageous phenotypes are selected and we might be observing only those IS*6110* transposition events providing benefits in terms of enhanced transmissibility or pathogenicity. In this context, those transposition events observed during our mouse infection experiments might be the result of enhanced fitness *in vivo*. Accordingly, serially infecting batch of mice with those bacteria resulting from transposition events would surely enrich the bacterial population for IS*6110* locations conferring selective phenotypes.

The transposition dynamics of IS*6110* imply an exponential relationship between the copy number content and mRNA expression per IS*6110* copy, (**Fig 2D**). Accordingly, the increased expression per IS*6110* copy observed in high copy number strains (**Fig 2C**) provide more messenger molecules and this probably results in increased probability of ribosomal frameshift and translation of a functional transposase, leading to accumulation of this mobile element across the chromosome. On the other hand, even if transposition generally occurs at random across the MTBC chromosomes, it remains to be answered why some genomic regions such as a 600Kb close to the origin of replication lack IS*6110*, pointing to the detrimental effect of insertions in this location [56], while other regions such as *plcD* are prone to accumulate IS*6110* insertions and result in IS*6110*-mediated deletions such as RvD2 [57].

Since only subgroups STB-A, -D and–L (but not–J and–K) of the MTBC progenitor *M*. *canettii* contain IS*6110*, we can hypothesize about the origin of this transposon prior to or during the evolution of the MTBC progenitor. Recent evidence has shown that *M*. *canettii* strains, in contrast to the MTBC, are not clonal and could exchange DNA [58]. *M*. *canettii* STB-D, -A and -L share adjacent C-term and N-term truncated regions of IS*6110* separated by 1,2 Kb (**S1 Fig**). DNA binding and integrase domains are located in the opposite ends of the IS*6110* coding sequence (**S5 Fig**) and we can hypothesize about the origin of IS*6110* by a recombination between these adjacent regions (**S1 Fig**). Reinforcing this hypothesis, similar recombination events leading to surface remodelling have been recently documented in *M*. *canettii* [59].

The low transposition frequencies observed during the natural infection support the remarkable value of IS*6110* as a molecular epidemiology marker. Transposition is probably maintained at low levels by the action of several mechanisms. Here, we found two regulatory pathways involving translational slippage or the formation of secondary RNA structures, such as pseudoknot, but we cannot discard other regulatory mechanisms. The putative ribosome binding sequence of the IS*6110* is occluded by a stem loop (**S5C Fig**) and formation of this structure is expected to have some impact over translation of the transposase. An important question is whether the mRNA initiates within the own IS*6110* or whether it initiates upstream from an adjacent promoter in the MTBC chromosome. In this latter case, IS*6110* transcription might very well depend on the precise location of this transposon within the host chromosome. This assumption would justify why high copy number strains are associated with higher expression rates per IS*6110* copy and *vice versa* (**Figs 1 and 2**). The exploration of *M*. *bovis* RNA-seq data indicates negligible transcription of the unique IS*6110* copy in this species with no presence of neighbour transcription start sites [60, 61]. The position of the IS*6110* copy in *M*. *bovis* is shared by most members of the MTBC and it is located within the Direct Repeats (DR) region of the CRISPR-Cas locus. Since this region is subjected to a complex post-transcriptional regulation involving RNA processing steps, this might explain the low expression of this IS*6110* copy. This is congruent with our expression data (**Fig 2**) and reinforces the hypothesis that lower transcription is likely associated with decreased probability of translational frameshift and consequently with low transposition rates in *M*. *bovis* (**Fig 5B and 5C**).

Our results with *M*. *tuberculosis* H37Rv indicate that transposition of IS*6110* is not limited to the natural infection and also occurs during growth *in vitro* (**Fig 5B**). Supporting this finding, the examination of H37Rv reference strains across multiple laboratories worldwide indicate different transposition events of IS*6110* [62]. Other example of changes in the IS*6110* pattern is the presence of 19 and 15 IS*6110* copies in H37Ra and H37Rv respectively. Since these strains arose during laboratory subculture of the original H37R strain in the 1930’s, differential IS*6110* are likely the result of separate and individual transposition events during *in vitro* passage. It is interesting to observe higher transposition frequencies in long-term cultures (**Fig 5B**), but it remains to be answered whether this is the result of cumulative transposition events during the growth curve or transposition increases as a consequence of starvation signals. Examination of the IS*6110* mRNA expression indicates a strong upregulation during starvation (**Fig 5C**) which could indicate the presence of yet unknown stimulating signals triggering IS*6110* mobilization. To rule out that differences in the mutation rate of starved bacteria influence the transposition frequency, we should have performed a fluctuation test or similar. Nevertheless, a previous work demonstrated similar mutation rates during latency and during active disease or in a logarithmic growing culture [63], which agrees with the low mutation rates observed for MTBC chromosomes [40].

Several lines of evidence support a possible role for *IS6110* during adaptation to different hosts. Diverse epidemiological studies have demonstrated that IS*6110* RFLP presents distinct profiles in *M*. *tuberculosis* transmission clusters [28, 64–67]. Since these studies involve isolates from different patients isolated during prolonged periods of time, it is plausible to think that IS*6110*-mediated adaptive mechanisms might be involved in the patient-to-patient transmission of *M*. *tuberculosis*. Supporting this idea, our results indicate higher transposition rates during infection (**Fig 5D**). Another evidence comes from the observation that *M*. *bovis* are able to infect humans but rarely transmits between this population. However, a specific *M*. *bovis* strain was responsible of a deathly human MDR TB outbreak [68, 69] and this phenotype is largely related to transposition of a second IS*6110* [70]. This second IS*6110* is located upstream the *phoPR* virulence genes and acts as a mobile exogenous promoter increasing virulence phenotypes in *M*. *bovis* [71]. A very recent study demonstrates that IS*6110*-mediated deletions in the *ppe38-ppe71* genes are widespread in “modern” Beijing strains. This genotype result in lack of secretion of PE_PGRS and PPE-MPTR proteins and lead to increased virulence. Accordingly, this specific deletion mediated by IS*6110* may have contributed to the success and global distribution of this Beijing sublineage [72]. A previous study confirmed that Beijing (L2) strains have higher mutation rates than L4 strains, which result in increased acquisition of drug resistance in the former [73]. It is at present unknown whether varying mutational rates across MTBC lineages can impact on transposition rates. Further work is needed to confirm whether higher mutation rates provide the driving force for increased transposition or *viceversa*.

In conclusion, our findings indicate that the lineage-specific number of IS*6110* results from differential transcriptional and posttranscriptional mechanisms inherent to the MTBC chromosomes in order to control the copy number of this transposon. Our results show that IS*6110* transposition increases during mouse infection and during growth in starvation suggesting the potential role of IS*6110* transposition during the MTBC adaptation to the host. In the future, many MTBC strains are being massively sequenced, this opportunity should be taken into consideration to locate the IS*6110* insertion sites, which would lead us to a better understanding of its biological role in TB pathogenesis and life cycle.

## Material and methods

### Ethics statement

All procedures were carried out under Project Licence PI14/14 approved by the Ethic Committee for Animal Experiments from the University of Zaragoza. The care and use of animals were performed accordingly with the Spanish Policy for Animal Protection RD53/2013, which meets the European Union Directive 2010/63 on the protection of animals used for experimental and other scientific purposes.

### MTBC strains, growth conditions and chemicals

Strains from the MTBC and *M*. *smegmatis* mc^2^155 were routinely grown at 37°C in 7H9 medium (Difco) supplemented with 0.05% Tween 80 and 10% albumin-dextrose-catalase (ADC, Middlebrook) or on 7H10 plates supplemented with 10% ADC. For MTBC strains different from *M*. *tuberculosis*, 40 mM sodium pyruvate was added to the medium. *E*. *coli* DH5α used for cloning procedures was grown at 37°C in LB broth or on LB agar plates. Ampicillin (100 μg/ml), kanamycin (20 μg/ml) and hygromycin (20 μg/ml) were used as appropriate. For transposition experiments, cultures were incubated at 30°C or 37°C and sucrose was added to 7H10 plates at a final concentration of 2% for *M*. *tuberculosis* and *M*. *bovis* and 10% for *M*. *smegmatis* if appropriate. All chemicals were purchased from Sigma-Aldrich, unless otherwise stated.

### Plasmid construction

IS*6110* containing IR was PCR amplified from *M*. *tuberculosis* H37Rv DNA using primers *Nhe*I-IS*6110*-fw (GCTAGCTGAACCGCCCCGGCATG) and *Nhe*I-IS*6110*-rv (GCTAGCTGAACCGCCCCGGTGAGT). The PCR product was digested with *Nhe*I and cloned into *Nhe*I cut pMV361 to yield pIS*6110*-WT. To construct IS*6110* carrying the -1 translational frameshift, a two-step overlapping PCR strategy was used. ORF1 was amplified with primers *Nhe*I-IS*6110*-fw and *IS6110*-FS-rv (CGACGCGGTCTTTTAAAATCGCGT) and ORF2 with *Nhe*I-IS*6110*-rv and *IS6110*-FS-fw (ACGCGATTTTAAAAGACCGCGTCG). Both PCR products overlap in 24 nucleotides and carry the extra nucleotide required for the translational frameshifting (underlined nucleotides). These products were used as self-templates in a PCR reaction that was amplified using the flanking primers *Nhe*I-IS*6110*-fw and *Nhe*I-IS*6110*-rv, digested with *Nhe*I and introduced in the *Nhe*I site of pMV361 to yield pIS*6110-*FS. The resulting constructs were confirmed by Sanger sequencing, introduced in *M*. *smegmatis* mc^2^155 by electroporation and colonies carrying a chromosome-integrated vector were checked by PCR.

To construct the transposition reporter, a kanamycin resistance cassette from pKD4 was amplified with primers *Bam*HI-IR1-P1 (cgcggatccgcgTGAACCGCCCCGGCATGTCCGGAGACTCgtgtaggctggagctgcttc) and *Bam*HI-IR2-P2 (cgcggatccgcgTGAACCGCCCCGGTGAGTCCGGAGACTCcatatgaatatcctccttag), which include the IR from IS*6110* indicated in capital letters. The PCR product was confirmed by Sanger sequencing, digested with *Bam*HI and introduced into pPR27 cut with the same enzyme. The final plasmid was named pIR-Km and was introduced into *M*. *smegmatis* mc^2^155, *M*. *tuberculosis* H37Rv and *M*. *bovis* AF2122/97 by electroporation. Transformants were selected with kanamycin at 30°C and cultured at this permissive temperature to allow plasmid replication.

Tagged variants of IS*6110* were obtained by gene synthesis (Genescript) as follows: a 3xFLAG epitope (DYKDHDGDYKDHDIDYKDDDDK) with codons optimized for *M*. *tuberculosis* was placed in frame immediately after the IS*6110* coding sequence. To construct the transcriptionally active IS*6110*-FS, an A insertion was placed after the Leu92 codon. To construct the IS*6110*-FS+PK variant carrying mutations disrupting the pseudoknot, the original sequence (cgcggccgagctcgaccggccagcacgctaattacccggttcatcgccgatcatcagggccaccgcgagggccccgatggtttgcggtggggtgtcgag) was replaced by (cgggggcgtgcacgtcccgcgagtacgctaattacgcggtttattgccgaccaccaagggcaccgcgaggggcccgacggcttaaggtggggagtggaa) to maintain the aminoacid sequence. The final constructs were flanked by *Xmn*I and *Eco*RI sites at the 5’ and 3’ ends respectively and cloned between these sites in pMV361. These plasmids were introduced in *E*. *coli* DH5α for subsequent experiments.

### Restriction fragment length polymorphism of IS*6110*

DNA from MTBC strains or *M*. *smegmatis* mc^2^155 were extracted by the CTAB/NaCl procedure. DNA integrity was confirmed by agarose gel electrophoresis. For standard IS*6110* RFLP, DNA was digested with *Pvu*II and separated overnight in 0.8% agarose gels. DNA was transferred from the gel to a positively charged nylon membrane (Hybond N^+^, Amersham) by using a vacuum transfer device. The membrane was hybridized with a probe amplified with primers INS-1 (CGTGAGGGCATCGAGGTGGC) and INS-2 (GCGTAGGCGTCGGTGACAAA). After hybridization with labeled DNA probes, the bound probes were detected with an enhanced chemiluminescence direct nucleic acid detection system (Amersham) according to the manufacturer's recommendations.

For RFLP of colonies resulting from transposition of the IR-Km-IR cassette from the pIR-Km transposition reporter, these modifications were introduced in the RFLP protocol: DNA was digested with *Pst*I and hybridized with a probe amplified with P1 (GTGTAGGCTGGAGCTGCTTC) and Km-pKD4-out1 (CCACGATAGCCGCGCTGCCTCG) primers using pIR-Km as template.

### Bioinformatic analysis

Genome sequences were retrieved from NCBI GenBank (http://www.ncbi.nlm.nih.gov/). The copy number content and genomic polymorphisms in IS*6110* were calculated using nucleotide BLAST (https://blast.ncbi.nlm.nih.gov/Blast.cgi).

Secondary RNA structures were predicted using the RNA fold WebServer (http://rna.tbi.univie.ac.at/cgi-bin/RNAWebSuite/RNAfold.cgi). Pseudoknot structures and their estimated free energy were located and computed using DotKnot (http://dotknot.csse.uwa.edu.au/).

### RNA extraction and normalized expression of IS*6110*

Mycobacterial cultures were grown to exponential phase (OD_600_ = 0.5–0.6) and pelleted by centrifugation. To minimize RNA degradation bacteria were resuspended in 1 ml RNA Protect Bacteria Reagent (Qiagen), incubated for 5 min at room temperature and then centrifuged. Bacterial pellets were resuspended in 0.4 ml lysis buffer (0.5% SDS, 20 mM NaAc, 0.1 mM EDTA) and 1 ml phenol:chloroform (pH = 4.5) 1:1. Suspensions were transferred to tubes containing glass beads (Qbiogene) and lysed using a Fast-prep instrument with a three-cycle program (15 sec at speed 6.5 m) including cooling the samples on ice for 5 min between pulses. Samples were then centrifuged and the homogenate was removed from the beads and transferred to a tube containing chloroform:isoamylalcohol 24:1. Tubes were inverted carefully before centrifugation and the upper (aqueous) phase was then transferred to a fresh tube containing 0.3 M Na-acetate (pH = 5.5) and isopropanol. Precipitated nucleic acids were collected by centrifugation. The pellets were rinsed with 70% ethanol and air dried before being re-dissolved in RNase-free water. DNA was removed from RNA samples using Turbo DNA free (Ambion) by incubation at 37°C for 1 h. RNA integrity was assessed by agarose gel electrophoresis and absence of contaminating DNA was checked by lack of amplification products after 30 PCR cycles.

One microgram of MTBC RNA was converted to cDNA using SuperScript III Reverse Transcriptase (Invitrogen) according to the manufacturer’s recommendations. The 10 μl PCR reaction consisted of 1X SYBR Green PCR Master Mix (Applied Biosystems), 0.25 μM of each primer and 1 μl of 1:10 diluted cDNA or IP DNA from immunoprecipitation reactions. Reactions were carried out in triplicate in an Applied Biosystems StepOnePlus^TM^ Sequence Detection System (Applied Biosystems) according to the manufacturer’s instructions. Melting curves were constructed to ensure that only one amplification product was obtained. Normalization was obtained to the number of *sigA* molecules in each sample. To obtain normalized expression values per IS*6110* copy number, data normalized with respect to *sigA* were subsequently divided by the total number of IS*6110* for every strain used. All qRT-PCR primers were designed using Primer Express software (Applied Biosystems) and sequences are as follows: RT-IS*6110*-1-fw (TCAGCACGATTCGGAGTGG), RT-IS*6110*-1-rv (CCAAGTAGACGGGCGACCT), RT-IS*6110*-2-fw (CGCAAAGTGTGGCTAACCCT), RT-IS*6110*-2-rv (GCATCTGGCCACCTCGAT), RT-sigA-fw (CCGATGACGACGAGGAGATC) and sigA-rv (CGGAGGCCTTGTCCTTTTC).

### Western blot

The pelleted fraction of bacterial cultures was resuspended in PBS containing 1% triton X100 and a cocktail of protease inhibitors (Roche) and disrupted using a Fast-Prep during three pulses, 1 minute each, cooling on ice between pulses. Samples were then centrifuged and the upper phase containing whole-cell lysate was quantitated using the RC DC protein assay (BioRad). Equal amounts of protein preparations were loaded per well. Proteins were separated on SDS-PAGE 12–15% gels and transferred onto PVDF membranes using a semidry electrophoresis transfer apparatus (Bio-Rad). Membranes were incubated in TBS-T blocking buffer (25 mM Tris pH 7.5, 150 mM NaCl, 0.05% Tween 20) with 5% w/v skimmed milk powder for 30 min prior to overnight incubation with primary antibodies at the dilution indicated below. Membranes were washed in TBS-T three times, and then incubated with secondary antibodies for 1 h before washing. Anti-FLAG (M2 clone, Sigma) antibody was used at 1:10,000 dilution and horseradish peroxidase (HRP) conjugated IgG secondary antibody (Sigma-Aldrich) was used at a 1:20,000 dilution. Signals were detected using chemiluminescent substrates (GE Healthcare).

### Mouse infection procedures

All mice were kept under controlled conditions and observed for any sign of disease. Experimental work was conducted in agreement with European and national directives for protection of experimental animals and with approval from the competent local ethics committees (approved protocol PI14/14). We performed a single biological replicate using 3 mice per group. Female C57BL/6 mice (Janvier Biolabs) were intranasally inoculated with 10^4^ CFU of *M*. *tuberculosis* H37Rv or *M*. *bovis* AF2122/97 (both carrying the transposition reporter). Infection was left to progress for 4 weeks and bacterial burden was determined by plating homogenized lungs and spleen on solid medium. Transposition events were enumerated as described in the “transposition experiments” section.

### Transposition experiments

Liquid cultures grown at 30°C or organ homogenates from infected mice were serially diluted and plated on 7H10 medium without sucrose at 30°C to enumerate viable bacteria. In parallel, appropriate dilutions were plated on 7H10 medium containing kanamycin and sucrose at 37°C to obtain colonies resulting from transposition of the IR-Km-IR cassette in the mycobacterial chromosome. The transposition frequency was calculated as the number of bacteria resulting from a transposition events divided by the number of total viable bacteria.

## Supporting information

S1 Fig IS*6110* in *M*. *canettii*. Network phylogeny of *M*. *canettii* sequenced strains adapted from [21]. The position of IS*6110* sequences in fully assembled genomes of *M*. *canettii* is indicated. Black dots indicate a wild type sequence, red dots indicate a mutated protein and red squares show positions of truncated IS*6110*. Only STB-D, STB-A and STB-L subgroups of *M*. *canettii* containing IS*6110* are indicated. The lower part of this figure indicate the positional arrangement of 5’ and 3’ terminus of the IS*6110* shared by STB-A, STB-D and STB-L and that of a functional IS*6110* in the remaining members of the MTBC. This organization suggest a recombination between both fragment in *M*. *canettii* to produce an active transposase.
(TIFF)

S2 Fig Normalised expression of ORF1 and ORF2. Blue and red bars indicate expression per IS*6110* copy for ORF1 and ORF2 respectively according to the left Y-axis. Red squares indicate the IS*6110* copy number n the analysed strains according to right Y-axis. Note that both constituent ORF from the transposase are equally expressed.
(TIFF)

S3 Fig IS*6110* gene expression in atypical copy number strains. (**a**) Normalised IS*6110* expression in low copy number *M*. *tuberculosis* strains. Note that *M*. *tuberculosis* containing a single IS*6110* expresses this gene at comparable levels to BCG Pasteur used as reference. Accumulation of further IS*6110* copies results in higher normalised expression values. (**b**) Normalised IS*6110* expression in “high copy” number *M*. *bovis* strains. Note that the presence of >1 IS*6110* copies in *M*. *bovis* results in high expression values compared to either BCG Pasteur used as reference or *M*. *tuberculosis* H37Rv. (**c**) Normalised IS*6110* expression in *M*. *tuberculosis* Lineage 2 (Beijing) strains. Note that normalised expression values are noticeable higher than those observed in *M*. *tuberculosis* H37Rv from lineage 4. (**d, e, f**) RFLP from MTBC strains analysed in panels a, b and c. Columns and error bars from panels (a), (b) and (c) are the standard deviation of the mean value from three independent cultures according to the left Y-axis. Red squares in panels (a), (b) and (c) indicate the IS*6110* copy number according to the right Y-axis.
(TIFF)

S4 Fig Expression of IS*6110* in the context of gene expression from diverse genes in *M*. *tuberculosis*. Each gene is measured relative to the *sigA* expression levels and columns indicate log10 values of normalised expression values. Note that IS*6110* expression per copy is within the range of genes producing physiological phenotypes such as *tatC* involved in protein secretion or *pks3* involved in acyltrehalose containing lipids.
(TIFF)

S5 Fig Genetic features and domain organization of the IS*6110* protein. (**a**) The two constituent ORF ara indicated by blue and red arrows. Position of transposase, integrase and helix-turn-helix domains are shown. The lower part of the panel include a description of the indicated domains. (**b**) The putative content of alpha-helices and beta-strands in the IS*6110* aminoacidic sequence is indicated by cylinders and arrows respectively (**c**) RNA secondary structure of the N-terminus. The RBS and the start codon are indicated by bold and underlined characters respectively. Note the presence of the stem loop occluding the RBS.
(TIFF)

S6 Fig Pseudoknot structure and mutational analysis. (**a**) Structure of the IS*6110* pseudoknot indicating the positions selected for mutation (asterisks). (**b**) Alignment of wild type and mutated variants of the pseudoknot. (**c**) Formation of secondary structures in the wild type and mutated variant indicating the ΔG values. Note the formation of a stable pseudoknot in the wild type but not in the mutated variant.
(TIFF)

S7 Fig Growth rates of liquid cultures at 30°C of *M*. *tuberculosis* or *M*. *bovis* transformed with pIR-Km are indicated blue and red lines respectively. Enumeration of CFU/mL represent the average and standard deviation from three independent cultures.
(TIFF)

S8 Fig IS*6110* expression during macrophage infection. Bars indicate normalised expression per IS*6110* copy after 4 and 24 hours of MHS macrophage infection (dark grey columns) relative to expression under laboratory growth (light grey columns). Data from two *M*. *tuberculosis* clinical isolates are provided. Results represent average and standard deviation from three independent infections.
(TIFF)
