# Supplementary figures and images for: New insights into the transposition mechanisms of IS6110 and its dynamic distribution between Mycobacterium tuberculosis Complex lineages
Source: PLoS Genet. 2018 Apr 12;14(4):e1007282. doi: 10.1371/journal.pgen.1007282 (PMC5896891; doi:10.1371/journal.pgen.1007282)

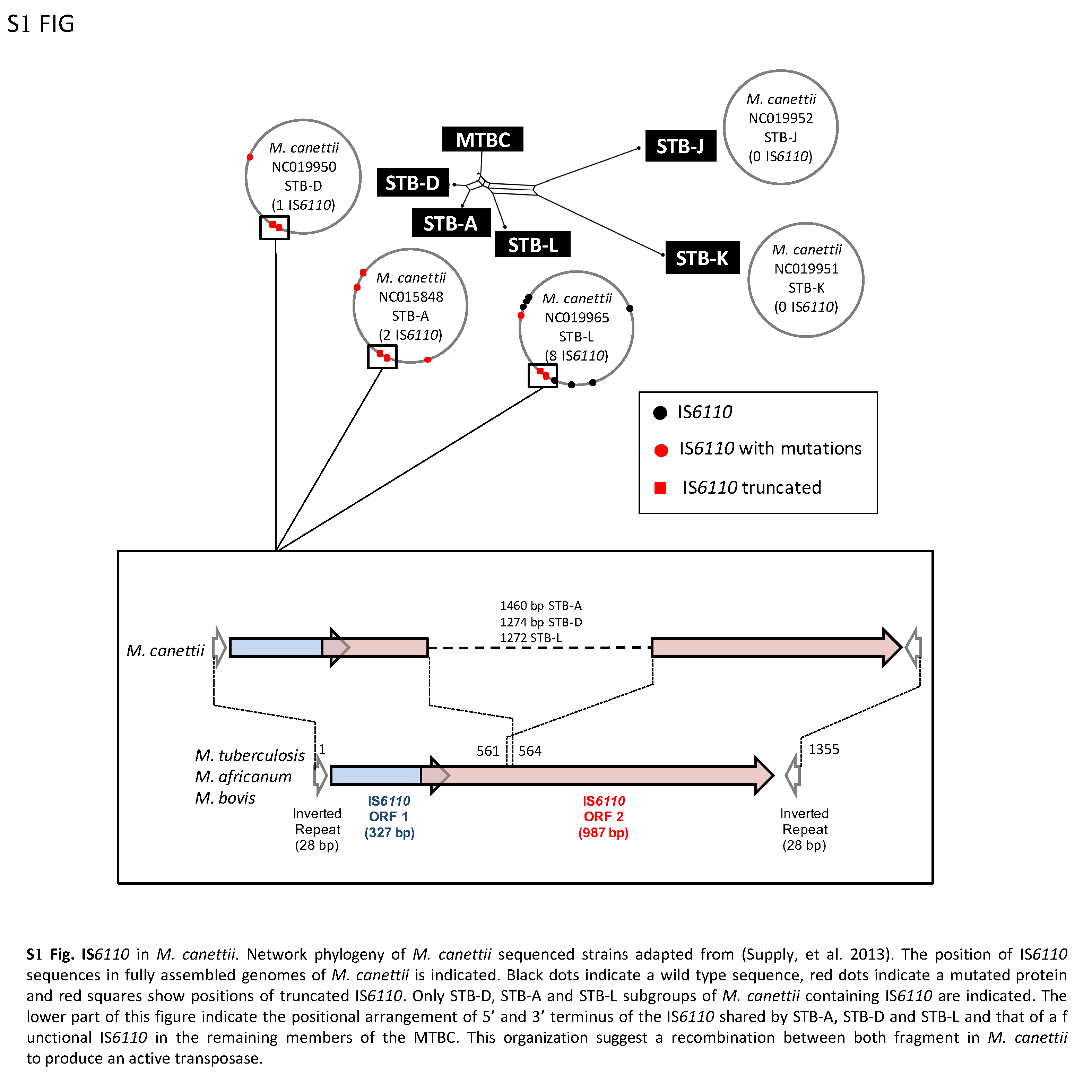

Supplement: S1 Fig — Network phylogeny of M. canettii sequenced strains adapted from [21]. The position of IS6110 sequences in fully assembled genomes of M. canettii is indicated. Black dots indicate a wild type sequence, red dots indicate a mutated protein and red squares show positions of truncated IS6110. Only STB-D, STB-A and STB-L subgroups of M. canettii containing IS6110 are indicated. The lower part of this figure indicate the positional arrangement of 5’ and 3’ terminus of the IS6110 shared by STB-A, STB-D and STB-L and that of a functional IS6110 in the remaining members of the MTBC. This organization suggest a recombination between both fragment in M. canettii to produce an active transposase. (TIFF) [file pgen.1007282.s001.tiff]

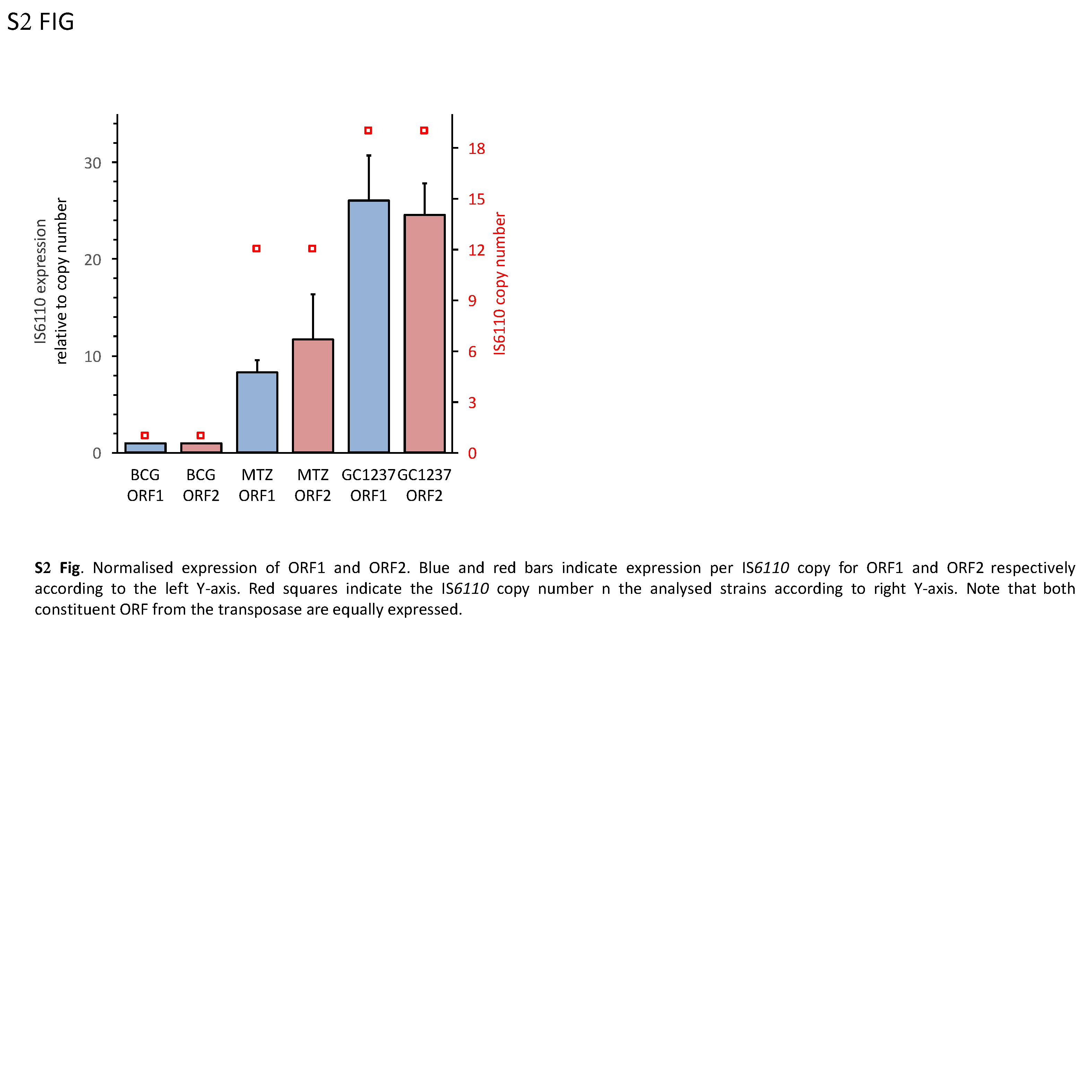

Supplement: S2 Fig — Blue and red bars indicate expression per IS6110 copy for ORF1 and ORF2 respectively according to the left Y-axis. Red squares indicate the IS6110 copy number n the analysed strains according to right Y-axis. Note that both constituent ORF from the transposase are equally expressed. (TIFF) [file pgen.1007282.s002.tiff]

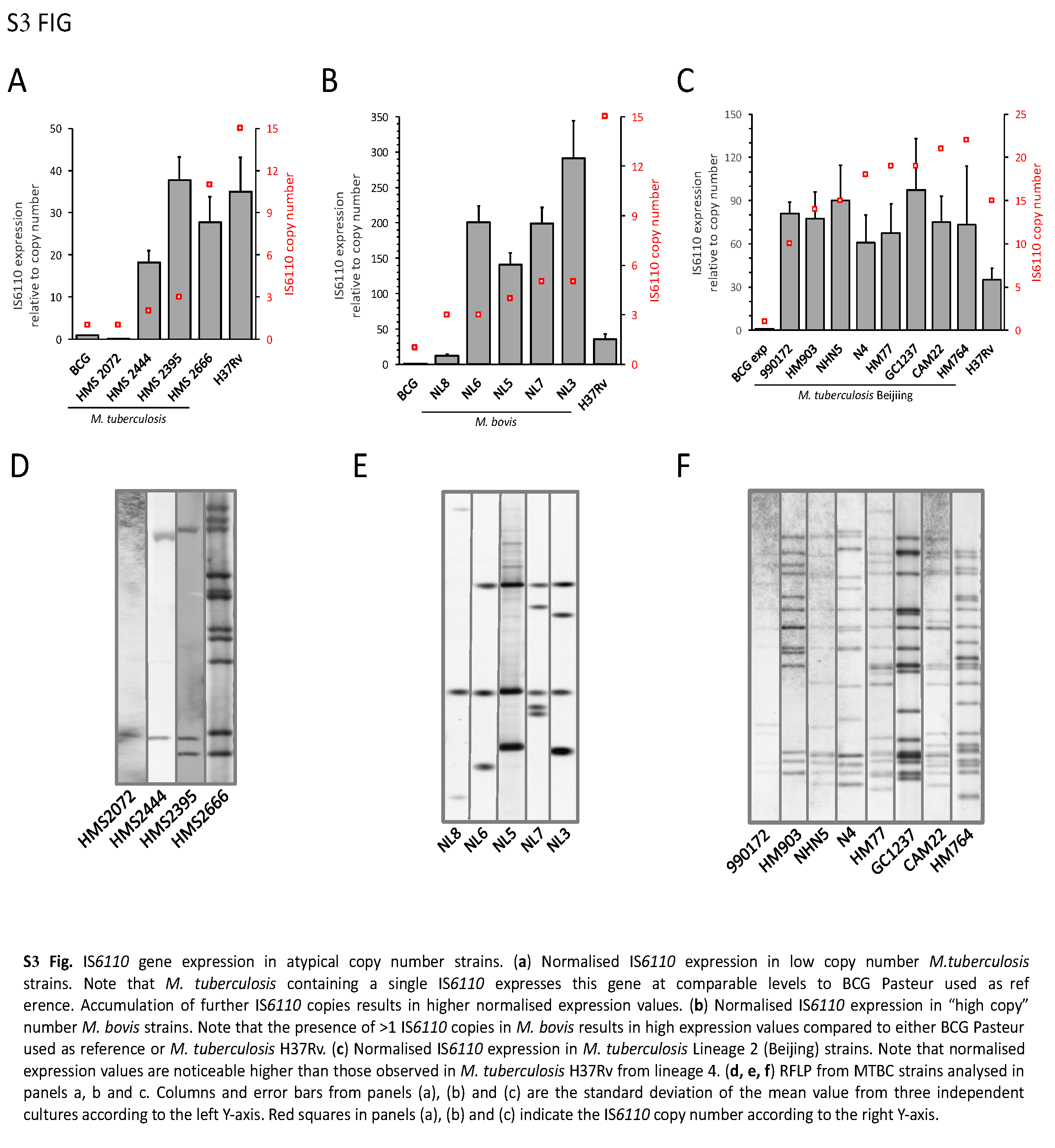

Supplement: S3 Fig — (a) Normalised IS6110 expression in low copy number M. tuberculosis strains. Note that M. tuberculosis containing a single IS6110 expresses this gene at comparable levels to BCG Pasteur used as reference. Accumulation of further IS6110 copies results in higher normalised expression values. (b) Normalised IS6110 expression in “high copy” number M. bovis strains. Note that the presence of >1 IS6110 copies in M. bovis results in high expression values compared to either BCG Pasteur used as reference or M. tuberculosis H37Rv. (c) Normalised IS6110 expression in M. tuberculosis Lineage 2 (Beijing) strains. Note that normalised expression values are noticeable higher than those observed in M. tuberculosis H37Rv from lineage 4. (d, e, f) RFLP from MTBC strains analysed in panels a, b and c. Columns and error bars from panels (a), (b) and (c) are the standard deviation of the mean value from three independent cultures according to the left Y-axis. Red squares in panels (a), (b) and (c) indicate the IS6110 copy number according to the right Y-axis. (TIFF) [file pgen.1007282.s003.tiff]

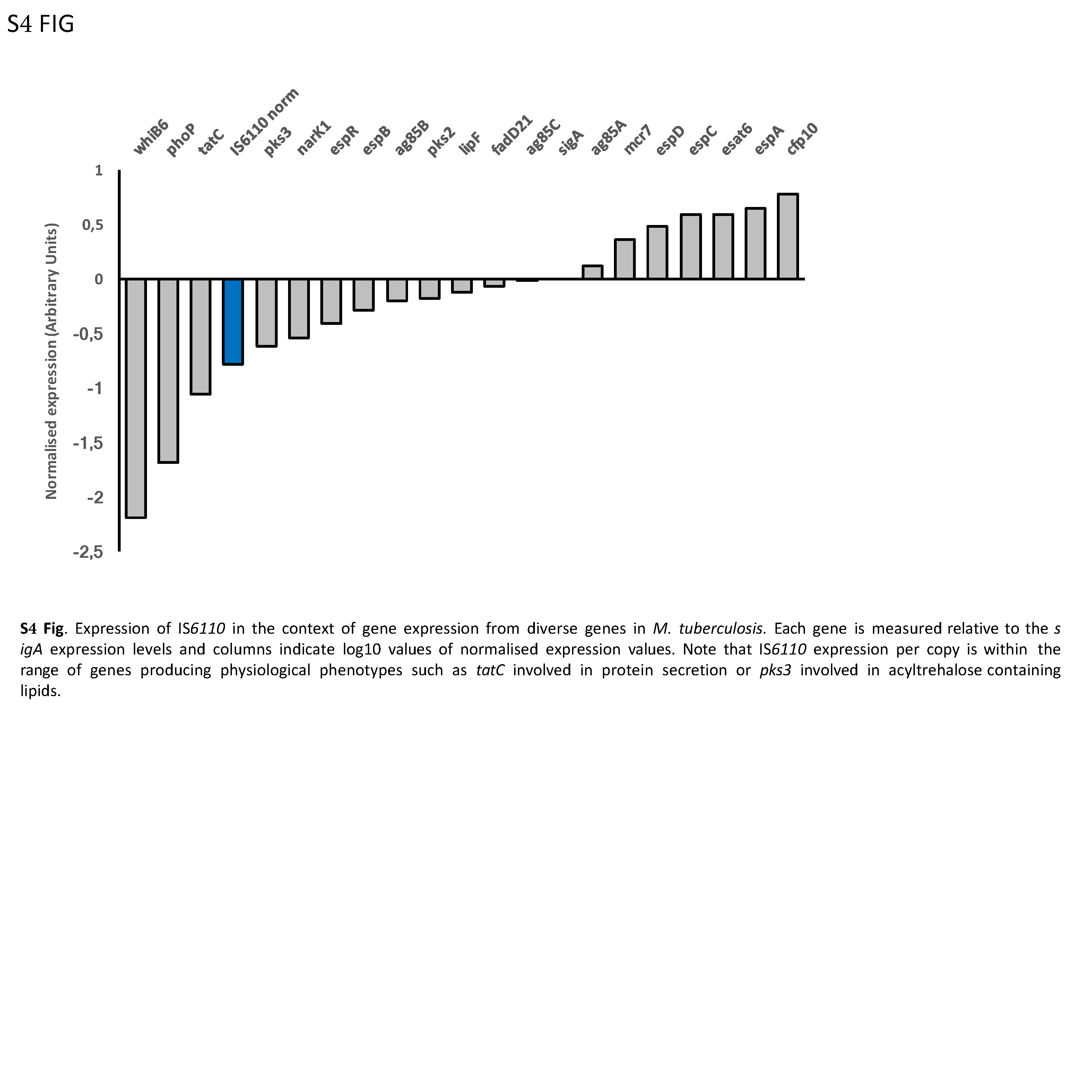

Supplement: S4 Fig — Each gene is measured relative to the sigA expression levels and columns indicate log10 values of normalised expression values. Note that IS6110 expression per copy is within the range of genes producing physiological phenotypes such as tatC involved in protein secretion or pks3 involved in acyltrehalose containing lipids. (TIFF) [file pgen.1007282.s004.tiff]

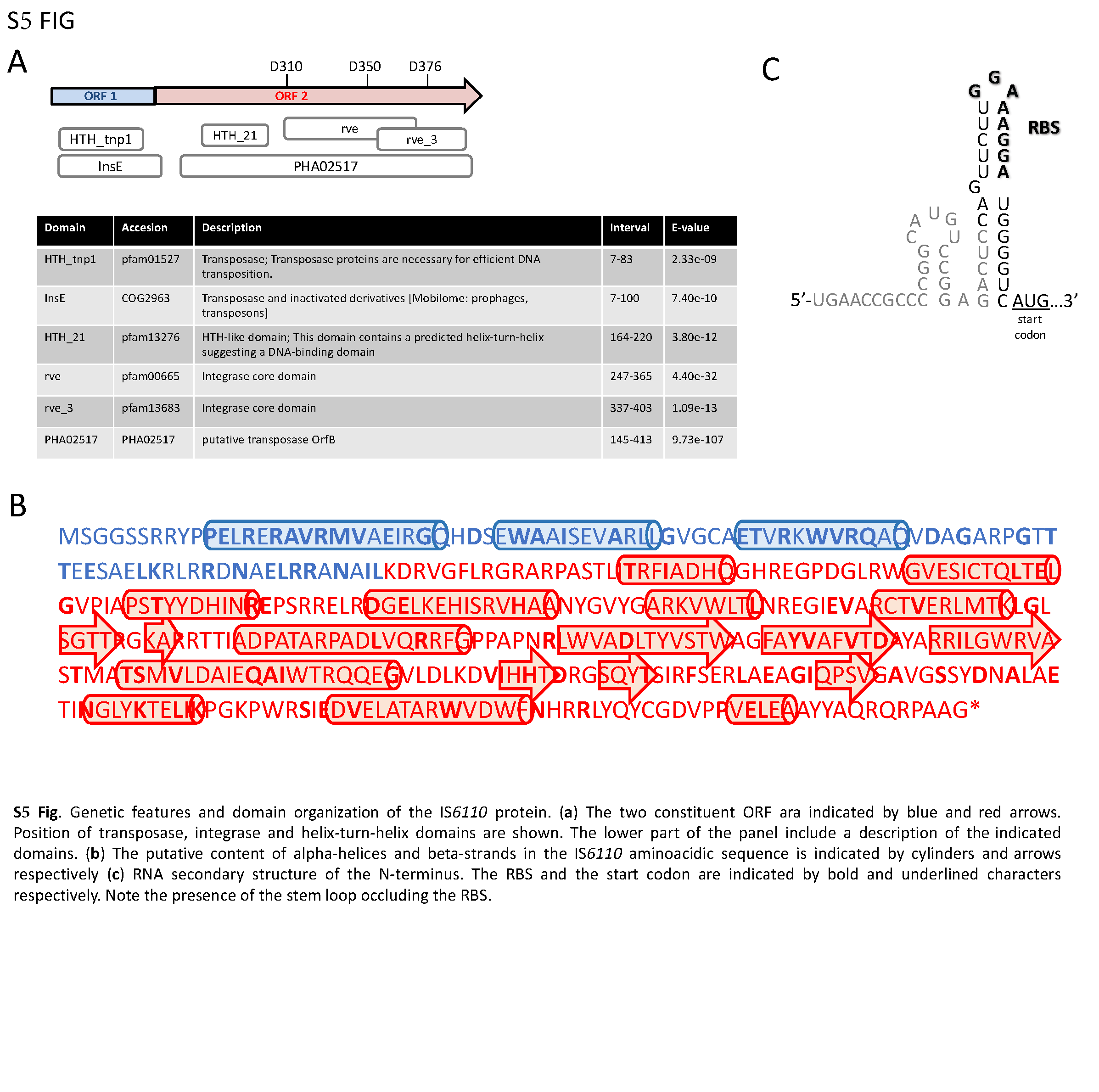

Supplement: S5 Fig — (a) The two constituent ORF ara indicated by blue and red arrows. Position of transposase, integrase and helix-turn-helix domains are shown. The lower part of the panel include a description of the indicated domains. (b) The putative content of alpha-helices and beta-strands in the IS6110 aminoacidic sequence is indicated by cylinders and arrows respectively (c) RNA secondary structure of the N-terminus. The RBS and the start codon are indicated by bold and underlined characters respectively. Note the presence of the stem loop occluding the RBS. (TIFF) [file pgen.1007282.s005.tiff]

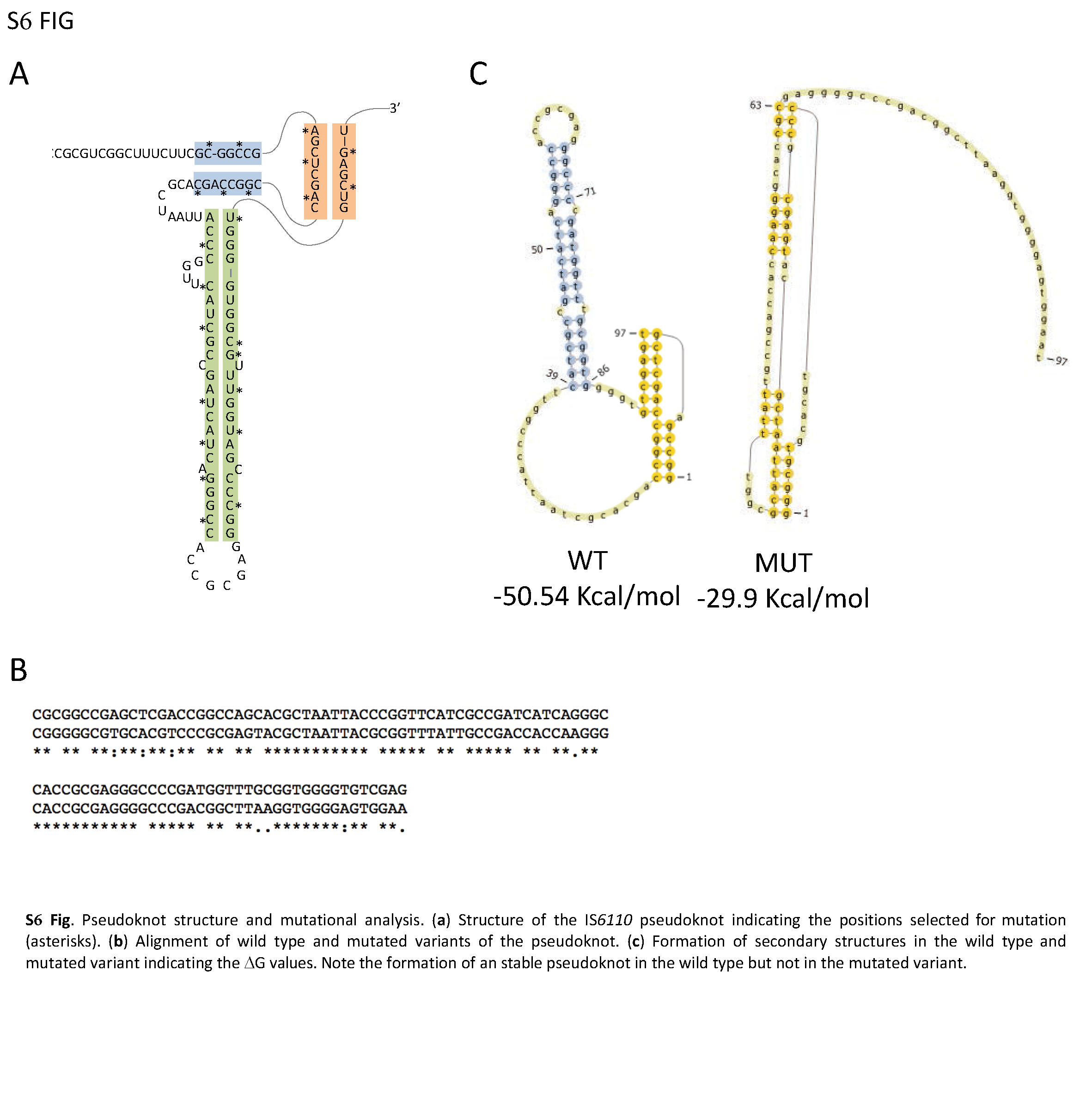

Supplement: S6 Fig — (a) Structure of the IS6110 pseudoknot indicating the positions selected for mutation (asterisks). (b) Alignment of wild type and mutated variants of the pseudoknot. (c) Formation of secondary structures in the wild type and mutated variant indicating the ΔG values. Note the formation of a stable pseudoknot in the wild type but not in the mutated variant. (TIFF) [file pgen.1007282.s006.tiff]

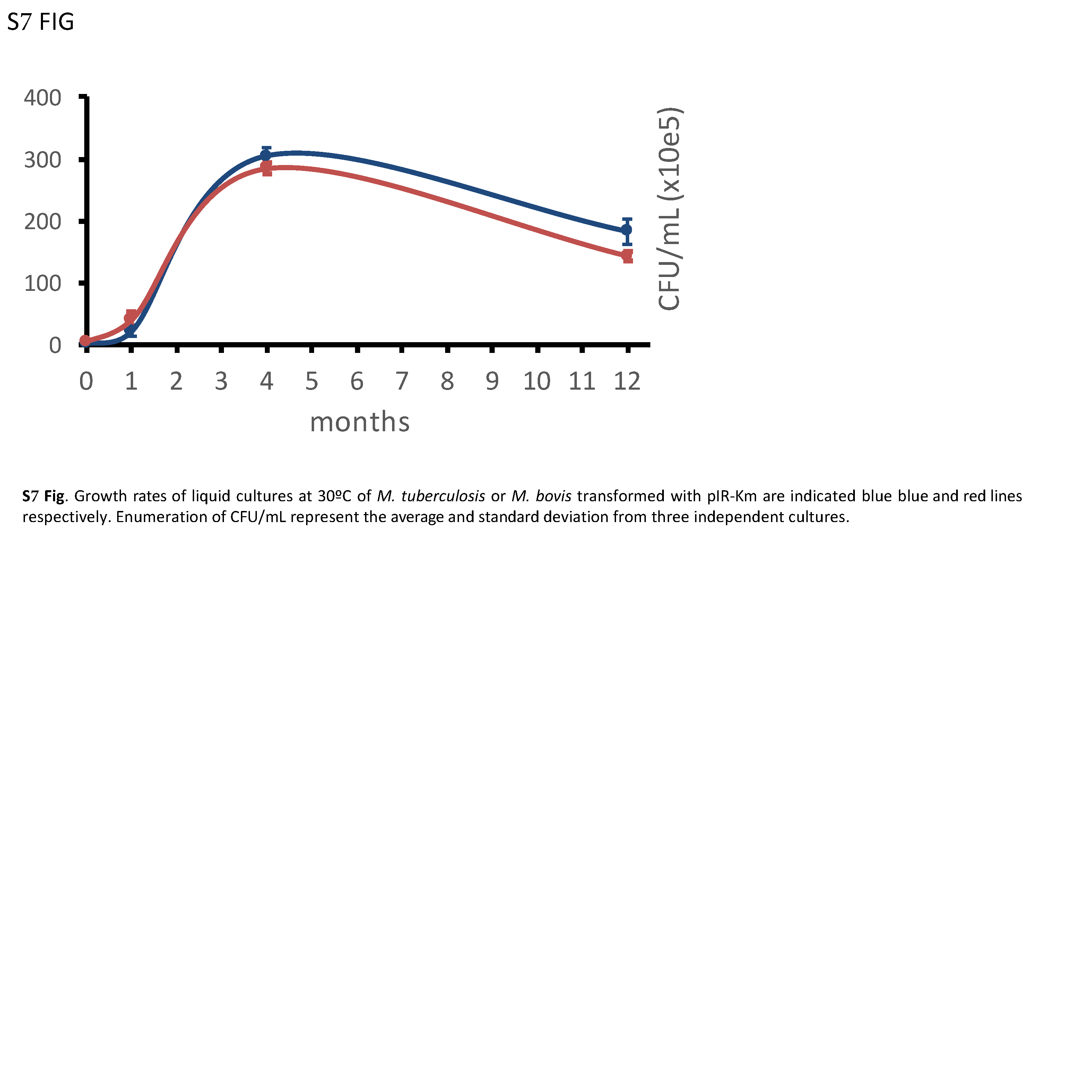

Supplement: S7 Fig — Enumeration of CFU/mL represent the average and standard deviation from three independent cultures. (TIFF) [file pgen.1007282.s007.tiff]

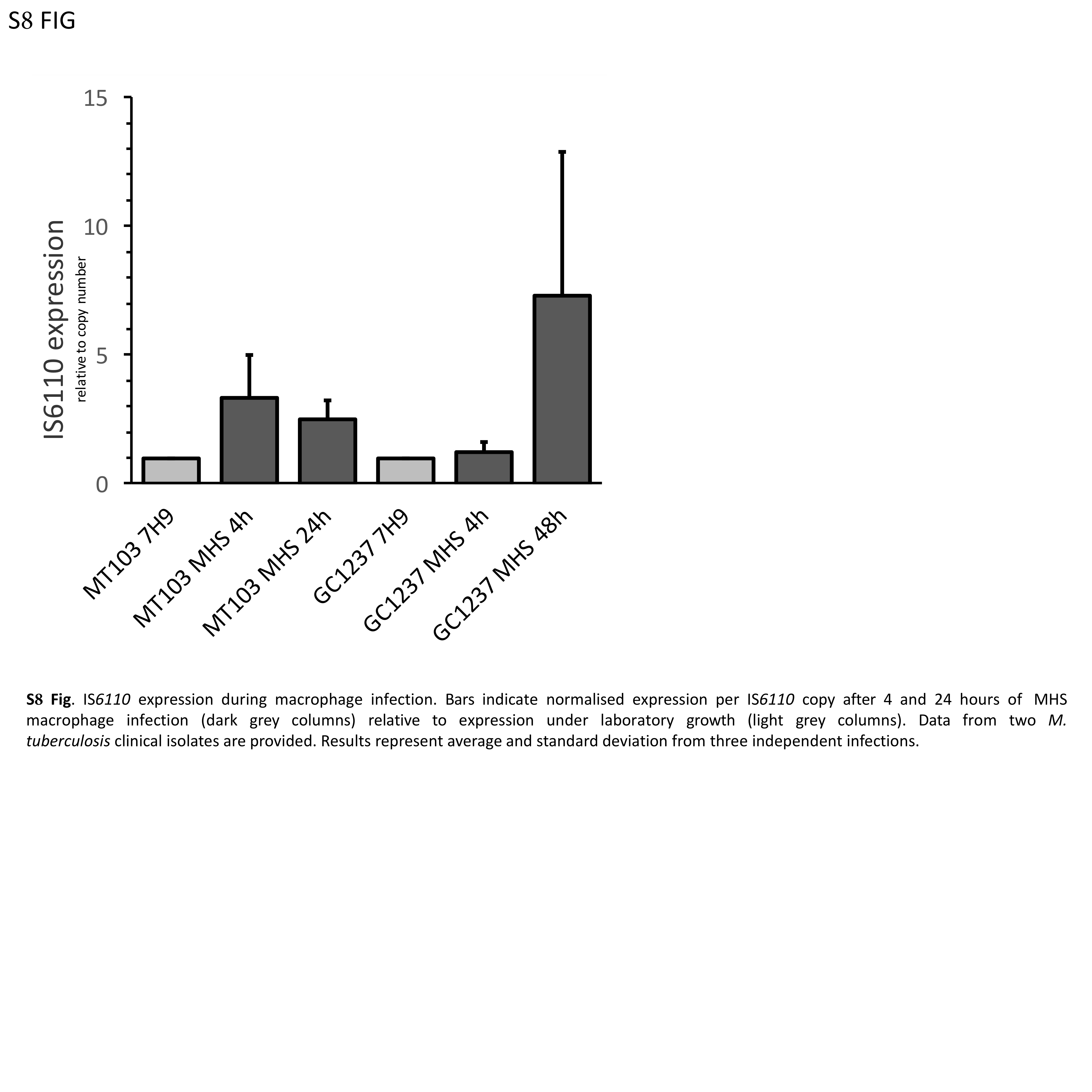

Supplement: S8 Fig — Bars indicate normalised expression per IS6110 copy after 4 and 24 hours of MHS macrophage infection (dark grey columns) relative to expression under laboratory growth (light grey columns). Data from two M. tuberculosis clinical isolates are provided. Results represent average and standard deviation from three independent infections. (TIFF) [file pgen.1007282.s008.tiff]
